# From zero to lab: Guidelines and practical implementation for building a multimodal experimental psychology and cognitive neuroscience laboratory

**DOI:** 10.3758/s13428-026-02988-0

**Published:** 2026-03-30

**Authors:** David del Rosario-Gilabert, A. García-Miquel, I. Vigué-Guix

**Affiliations:** Instituto de Neurociencia Avanzada de Barcelona (INAB), Passeig del Mare Nostrum, 15, 08039 Barcelona, Spain

**Keywords:** Cognitive neuroscience, Neuroimaging, Multimodal integration, Laboratory design, Experimental reproducibility

## Abstract

As multimodal approaches become increasingly common in cognitive neuroscience, there is a growing need for clear guidelines for designing and building laboratories. Here, we present a structured framework for the development of a multimodal neuroscience laboratory, covering key aspects such as space selection, acoustic treatment, electromagnetic shielding, environmental control, data synchronization, and data management. We also describe its practical implementation at the Instituto de Neurociencia Avanzada de Barcelona (INAB), where the facility was configured to support both individual and multi-participant studies using electroencephalography (EEG), functional near-infrared spectroscopy (fNIRS), eye-tracking, and peripheral biosensors. The laboratory was designed to preserve signal quality, minimize artifacts, and promote experimental reproducibility through controlled environmental conditions and rigorous synchronization procedures. Validation tests confirmed that the recording space achieved reverberation times below 0.6 s and reduced electromagnetic interference (EMI) by more than 30 × . Taken together, these technical and practical considerations provide a replicable model that other research groups can adopt to improve standardization and data reliability in multimodal neuroscience settings.

**Supplementary Information:**

The online version contains supplementary material available at 10.3758/s13428-026-02988-0.

## Introduction

Over the past century, neuroscience has changed substantially as methodological innovations have progressively expanded our ability to quantify brain activity. A crucial milestone occurred in 1924, when Hans Berger recorded the first human electroencephalogram (EEG), providing access to electrical population activity with millisecond temporal resolution (Tudor et al., 2005). This development established the basis for studying neural oscillations, perceptual and cognitive processes, and a wide range of clinical phenomena. In the following decades, the field witnessed the emergence of additional neuroimaging techniques, including magnetoencephalography (MEG) in the late 1960 s (Cohen, 1968), functional magnetic resonance imaging (fMRI) in the early 1990 s (Kwong et al., 1992), and functional near-infrared spectroscopy (fNIRS) shortly thereafter (Villringer & Chance, 1997). Together with peripheral biosensors such as electrocardiography (ECG) and galvanic skin response (GSR), all these technologies have expanded the measurement scope of neuroscience, enabling the study of brain function across complementary spatial and temporal scales.

Each research technique has advanced neuroscience by contributing unique strengths. For example, EEG has been widely used to study neural oscillations in perception (e.g., Luck, 2014), attention (e.g., Hillyard et al., 1998), cognition (e.g., Ismail & Karwowski, 2020), and consciousness (e.g., Del Rosario-Gilabert & Vigué-Guix, 2025) with key applications such as brain–computer interfaces (BCI; e.g., Vigué-Guix & Soto-Faraco, 2023). fNIRS has allowed researchers to measure hemodynamic responses in real-world settings, offering a portable alternative to fMRI for studying cognitive load, language processing, and emotional states (e.g., Pinti et al., 2015; Quaresima & Ferrari, 2019). Eye-tracking has provided key insights into visual attention, reading, and decision-making, playing an important role in research on conscious perception and cognitive load (e.g., Duchowski, 2017). Peripheral biosensors—such as GSR, heart rate variability (HRV), and respiratory frequency—have been employed to assess emotional arousal, stress responses, and autonomic regulation, contributing to studies on affective neuroscience, consciousness, and mind–body interactions (e.g., Altıntop et al., 2020; Liuzzi et al., 2023; Schwerdtfeger et al., 2020). Lastly, hyperscanning has allowed the simultaneous recording of brain activity across multiple individuals, facilitating research on social and collective consciousness through neural synchronization and interpersonal dynamics (e.g., Hamilton, 2021).

The complementarity of these techniques has motivated a growing shift toward multimodal experimental designs by combining the strengths of each method while compensating for their limitations. EEG offers high temporal precision but limited anatomical specificity, whereas fNIRS and fMRI provide superior spatial resolution at slower temporal scales. Eye-tracking monitors saccades, fixations, and pupil dilation, offering behavioral and attentional markers that complement neural recordings. Peripheral biosensors provide autonomic nervous system indices (e.g., HRV and GSR), which are critical for studying cognitive and emotional states. Integrating these modalities allows researchers to examine how neural, physiological, and behavioral processes interact dynamically. This approach has been adopted in several domains: EEG-fMRI for exploring the neural correlates of cognitive states by linking real-time electrophysiological activity with deep-brain hemodynamic responses (Debener et al., 2006), EEG-fNIRS for combining oscillatory activity and slow hemodynamic changes during cognitive load and executive function tasks (Keles et al., 2016), and EEG-eye-tracking for aligning neural signals with gaze behavior in visual attention and reading (Dimigen et al., 2011). However, combining multiple modalities introduces challenges related to equipment interference, synchronization accuracy, and environmental constraints that can compromise data quality. These issues highlight the need for standardized guidelines for designing and operating multimodal neuroscience laboratories.

The present work addresses this gap by providing a structured framework for designing, implementing, and optimizing a multimodal neuroscience laboratory, together with a practical real-world application. We describe the development of a dedicated multimodal laboratory at the Instituto de Neurociencia Avanzada de Barcelona (INAB) in Spain, configured to support the advancement of empirical research in cognitive neuroscience. The laboratory integrates EEG and fNIRS for monitoring cortical activity, along with eye-tracking, peripheral biosensors, and hyperscanning techniques, offering a flexible and scalable platform for both individual and multi-participant experiments. Although future extensions may incorporate technologies such as transcranial magnetic stimulation (TMS), fMRI, or MEG, these fall outside the scope of the current work.

A limiting factor in the field is the scarcity of systematic references detailing how multimodal laboratories should be designed to ensure methodological consistency. Aside from the guidelines proposed for undergraduate teaching environments (Ledwidge et al., 2018), we found no comprehensive resources addressing advanced research settings, to the best of our knowledge. Recent work has focused on specific configurations or methodological improvements, such as the EEG-eye-tracking co-registration in infants (Kulke, 2025) or general frameworks to improve methodological precision and reproducibility in human neuroscience (Nebe et al., 2023). However, these contributions do not address the technical, architectural, and environmental constraints inherent to a fully integrated multimodal laboratory.

Here, we extend previous contributions by providing a holistic design approach that encompasses infrastructure (e.g., acoustic and electromagnetic optimization), hardware interoperability, data synchronization, and reproducibility standards. The paper is organized into two sections. First, we describe the design principles guiding the construction of a multimodal neuroscience laboratory, including location selection, room layout, acoustic attenuation, electromagnetic shielding, electrical grounding, environmental regulation (temperature, humidity, ventilation, and air quality), and lighting optimization. We also outline guidelines for equipment integration, synchronization procedures, and data management practices aligned with ethical and security requirements. These elements serve as a reference for optimizing data integrity, minimizing methodological variability, and improving the reproducibility of psychology and cognitive neuroscience experiments. Second, we report the practical implementation of these guidelines at INAB and provide quantitative assessments of acoustic treatment, electromagnetic shielding, environmental stability (temperature, humidity regulation, air quality control), lighting conditions, and synchronization accuracy. We also describe challenges encountered during installation and the solutions adopted to ensure high-quality biosignal acquisition. By documenting this process, we aim to support researchers in establishing reliable and reproducible multimodal research environments.

## Guidelines for designing a multimodal neuroscience lab

Designing a neuroscience laboratory for cognitive research requires a systematic approach to ensure high-quality data acquisition, participant and researcher comfort, and reliable multimodal integration. Signal quality is strongly influenced by the physical environment, making a high signal-to-noise ratio (SNR) crucial to ensure recordings reflect experimental manipulations rather than external noise sources. Uncontrolled factors—such as poor recording conditions, electrical interference, or unstable room environments—can substantially degrade SNR, and eye-tracking accuracy can be affected by inappropriate illumination. For reliable and reproducible measurements, it is also important to minimize participants’ allostatic load and reduce exposure to external distractions. Careful planning is therefore critical when configuring a multimodal facility. This section outlines key considerations for laboratory design, including infrastructure, environmental controls, and technological requirements for advanced cognitive neuroscience research. We also describe the methods, equipment, and technical specifications selected to optimize laboratory performance.

### Laboratory design and infrastructure modifications

#### Location and layout

##### Location

The location of a multimodal neuroscience laboratory plays a central role in ensuring adequate signal quality, participant comfort, and efficient workflow. Physical placement should minimize both acoustic and electromagnetic interference while providing a stable and controlled environment for testing. Ideally, the laboratory should be situated away from high-traffic areas and common noise sources (e.g., elevators, kitchenettes, hallways) and distanced away from electromagnetic contaminants such as server rooms, telecommunications antennas, or industrial equipment. Acoustic buffering or dedicated isolation materials may be required to mitigate environmental noise further and reduce electromagnetic contamination, particularly for sensitive biosignal recordings such as EEG and fNIRS.

##### Layout

The internal layout should support smooth experimental flow, minimize interference, and facilitate efficient movement for both participants and researchers. Adequate space for equipment organization, storage, and experimental flexibility is essential for uninterrupted operation. Participant comfort is also an important consideration, as excessive discomfort can negatively influence physiological responses and cognitive performance (Zhu et al., 2020).

Room size must be sufficient to accommodate multiple participants during hyperscanning studies while allowing space for equipment, storage, and researcher mobility. A two-room configuration separating the recording and control areas is strongly recommended,^1^ allowing researchers to monitor the session in real time without disturbing participants (Ledwidge et al., 2018). Although a visual window may be included, real-time communication is more effectively maintained using wired audio and video systems (microphones, speakers, cameras), which avoid unnecessary distractions and remain compatible with electromagnetic shielding requirements discussed later.

A dedicated waiting area should also be provided to allow participants to rest before the experiment begins, helping to reduce stress, stabilize attentional and physiological states, and improve readiness for cognitive tasks through the restorative effects of brief rest periods (Ginns et al., 2023). Taken together, these considerations support participant well-being while enhancing the reliability of collected data.

##### Practical considerations

In addition to spatial layout, several infrastructure components are necessary to support efficient laboratory operations and maintain an organized workspace. In the control room, two separate workstations with computers are recommended—one for real-time data acquisition and another for offline processing and analysis. Multiple monitors should be available to allow simultaneous visualization of different data streams, such as EEG/fNIRS signals, participant video feeds, and experiment logs. This arrangement enables researchers to detect equipment issues early and intervene when necessary.

Movable trolleys should be used to store and transport essential equipment—amplifiers, power supplies, electrodes, and consumables—allowing flexible positioning of devices during setup. Furniture in both rooms should be comfortable and neutral in appearance, with non-reflecting, light-absorbent surfaces to minimize visual distraction. A nearby sink is required for cleaning electrodes, consumables, and equipment, and for allowing participants to wash after the session. Access to laundry facilities is beneficial for cleaning towels, lab coats, and other materials that must remain hygienic for participant use. Implementing these practical considerations ensures smooth laboratory operation, enhances participant comfort, and supports high-quality biosignal acquisition.

#### Acoustic treatment

Effective acoustic conditioning is essential for maintaining signal integrity in auditory experiments and for ensuring participant comfort in cognitive neuroscience laboratories. An appropriate acoustic environment requires both sound isolation—preventing external noise from entering the recording room—and acoustic conditioning, which reduces internal reflections and improves overall listening conditions. In addition to choosing a location distant from major noise sources, structural acoustic treatments and internal noise management measures must be implemented to achieve consistent acoustic performance.

Recording rooms should incorporate high-performance soundproofing materials to limit airborne and structural noise transmission. Dense, absorptive treatments applied to walls, floors, and ceilings help block external intrusion, and floating floors are recommended to prevent vibration transfer from the building structure. All joints, seams, and openings must be acoustically sealed to avoid leakage paths. Double-layer wall assemblies and acoustic doors with high sound transmission class (STC) or sound reduction index (SRI) ratings further enhance isolation and reduce susceptibility to unwanted auditory interference (ASTM E2235-04, 2020; ISO 717–1:2020, 2020)*.*

Structural isolation must be complemented by internal acoustic treatments designed to control room eigenmodes, sound reflections, and reverberation phenomena. Excessive reverberation can degrade speech intelligibility and compromise auditory tasks. Installing sound-absorbing panels on walls and ceilings is an effective way to limit reflections and enhance sound clarity (Cox & D’Antonio, 2009). Additional devices such as Schroeder diffusers may be positioned strategically to improve sound field uniformity (Fujiwara et al., 2000).

Attention should also be paid to noise originating from heating, ventilation, and air conditioning (HVAC) systems, as well as laboratory equipment. HVAC units should operate quietly and, where possible, be acoustically decoupled from the main building infrastructure to reduce transmitted noise. Vibration mounts and flexible duct connectors help mitigate mechanical coupling (Bujoreanu & Benchea, 2016). Selecting low-noise laboratory equipment ensures that background sound levels remain minimal during recordings, supporting cleaner and more reliable data acquisition.

#### Electrical grounding protection

Electrical grounding is fundamental for ensuring participant safety, minimizing signal interference, and maintaining high-quality EEG recordings. A stable ground reduces internal electrical noise and protects equipment against power surges or voltage fluctuations. Grounding alone, however, cannot eliminate external electromagnetic interference, which must be addressed through complementary shielding strategies described in later sections. Effective grounding requires that all power outlets in the laboratory include a dedicated grounding connection, typically provided by the third pin in standard outlets. This connection offers a return path through the neutral line and is linked to a conductive rod or equivalent structure embedded in the ground, forming the “earth ground” (Ledwidge et al., 2018).

Beyond standard electrical grounding, additional steps are needed to optimize EEG recordings and achieve a high SNR (Usakli, 2010). Metal surfaces in the laboratory—such as equipment racks, workstations, and structural fixtures—should be properly grounded to reduce power line noise. Separating analog and digital grounds is also critical to prevent interference between low-amplitude biosignals and noise generated by computers or other digital equipment. Grounding integrity should be checked regularly to ensure stable electrical performance and to detect faults that may compromise data quality.

Further measures can help reduce electrical interference during recordings. DC-powered lighting is preferable to AC lighting, as AC sources generate low-frequency fluctuations that can contaminate EEG signals. When AC lighting cannot be avoided, fixtures should be positioned at a distance from the participant to minimize interference (Ledwidge et al., 2018). Additionally, placing EEG and other biosignal acquisition systems on isolated power circuits reduces the likelihood of cross-interference from unrelated electrical devices operating in the laboratory.

#### Electromagnetic shielding

Electrical grounding reduces internal electrical noise, but additional electromagnetic shielding is required to limit interference from external sources such as power lines, electronic devices, and radio-frequency emissions. Electromagnetic interference (EMI) is a well-known contaminant in EEG recordings and can markedly degrade signal quality by introducing noise and artifacts that compromise data reliability (Jiang et al., 2019). Although common EMI contributors—power line noise and general electrical equipment—are widely recognized, less obvious sources such as audio and video systems, lighting fixtures, Wi-Fi routers, and mobile phones can also introduce high-frequency interference or oscillatory artifacts, especially when located near the EEG setup (see Table 1). Power line artifacts (50/60 Hz) and other narrowband signals can often be attenuated during preprocessing (Sweeney et al., 2012), but shielding remains the primary means of preventing contamination before it reaches the recording system. Importantly, filters cannot fully eliminate broadband or nonstationary noise components, meaning that EMI mitigation at the environmental level is essential for ensuring high-quality biosignal recordings (Sweeney et al., 2012).

**Table 1:** Common sources of electromagnetic interference (EMI) in neuroscience laboratories and their impact on EEG recordings

EMI source	Frequency band	Impact level	Impact description
Power lines	50/60 Hz	High	Significant 50/60 Hz artifacts, power line interference
Broadcast radio frequencies (AM/FM)	535–1705 kHz (AM) 88–108 MHz (FM)	Low-to-moderate	Can introduce low to moderate signal distortions depending on proximity
Broadcast TV	54–806 MHz (VHF/UHF)	Low	Minimal impact unless strong nearby sources are present
Mobile phones	700 MHz – 3.7 GHz (4G), 24–52 GHz (5G)	Moderate	Periodic signal interference, especially in low-frequency EEG bands
Wi-Fi routers	2.4 GHz, 5 GHz, 6 GHz	Moderate-to-high	High-frequency interference, affects wireless EEG systems
Bluetooth devices	2.4 GHz	Moderate	Moderate interference, especially if close to EEG equipment
General electrical equipment	Varies depending on the equipment	Varies	Can generate electrical noise and signal artifacts
Other high-frequency transmissions	1 GHz – 300 GHz	Depends on frequency and proximity	Potential for interference depending on specific transmission
Medical devices (e.g., MRI, TMS)	Varies (low kHz to high MHz)	High	Strong magnetic fields can completely distort EEG signals
Neon and fluorescent lighting	20–60 kHz	Moderate	Produces oscillatory noise and harmonics, interfering with EEG signals
HVAC systems and motors	50 Hz – 1 kHz	Moderate	Motors and HVAC units can introduce low-frequency noise

Summary of common EMI sources, their frequency bands, impact levels, and effects on EEG signal quality. The table highlights potential signal distortions, artifacts, and noise contamination introduced by environmental and electronic sources, emphasizing the need for mitigation strategies to ensure high-quality EEG recordings.

##### Recommended electromagnetic shielding strategies

Several strategies can be implemented to reduce EMI and maintain a clean recording environment. Prior work highlights the importance of shielding amplifier housings and electronic components with metal casings, minimizing the use of interference-prone devices such as fluorescent lighting (Usakli, 2010), and employing twisted and shielded cables for EEG signal transmission (Luck, 2014; Teplan, 2002). Instrumentation amplifiers with a high common-mode rejection ratio (CMRR > 80 dB) are recommended to suppress common-mode noise, although amplifier choice must balance noise rejection with potential trade-offs—such as improper grounding—that may introduce additional artifacts.

One of the most effective approaches to EMI control is the installation of a Faraday cage, a conductive enclosure that blocks external electromagnetic fields from reaching sensitive recording equipment. Converting a room into a functional Faraday cage requires covering walls, ceilings, floors, and doors with conductive materials such as conductive paint, copper mesh, aluminum foil, conductive fabric, or galvanized steel sheets. Multiple layers of conductive paint can serve as a cost-effective solution, with thicker or more conductive coatings providing optimal shielding. Ventilation ducts must be fitted with EMI-shielding panels to maintain airflow while preserving the cage’s integrity. All shielding materials must be electrically grounded to dissipate incoming electromagnetic signals effectively.

Beyond structural modifications, several laboratory-specific practices can further strengthen EMI protection. Limiting openings for cables and personnel reduces potential leakage paths, and sealing all remaining gaps with conductive materials maintains shielding continuity. Wireless devices should not be used inside a Faraday cage, as internal resonance can amplify rather than suppress electromagnetic fields if the cage geometry aligns with specific wavelengths (Hewett & Hewitt, 2016). Applying electromagnetic absorber materials to interior surfaces helps prevent high-frequency reflections that create localized EMI “hot spots.” Wired communication should be prioritized over wireless alternatives, and EMI filters or ferrites on power and signal cables can further suppress high-frequency noise. Transformers and lighting-related electronics should be enclosed in metallic shielding to reduce interference.

Once shielding has been installed, verification and maintenance procedures are necessary to ensure continued effectiveness. EMI measurements should be conducted using appropriate electromagnetic field detectors—for example, the MK70-3D Plus 2.2 from Gigahertz, which covers 5 Hz to 10 GHz. Over time, shielding performance may degrade due to physical wear or structural changes, particularly in high-use environments. Periodic inspections should therefore be performed to identify and repair breaches. Compliance with relevant local and international regulations governing electromagnetic shielding and electrical safety is required to maintain laboratory operational standards.

#### Environmental controls

Careful regulation of environmental factors such as temperature, humidity, and air ventilation is essential for maintaining participant comfort, ensuring equipment stability, and preserving biosignal quality. Fluctuations in these variables can introduce noise into recordings, alter electrode performance, and affect cognitive functioning, ultimately reducing data reliability.

##### Temperature regulation

Maintaining a stable laboratory temperature between 20 and 22 °C is recommended, as deviations can influence both participant physiology and the performance of sensitive electronics (Wang et al., 2023; Zhu et al., 2020). Temperatures above 23 °C have been associated with reduced cognitive performance and increased mental workload, reflected in characteristic EEG activity changes (Yeom et al., 2017; Zhu et al., 2020). Even moderate thermal variations can modify EEG frequency bands and power spectra, potentially affecting experimental outcomes (Deboer, 1998). Temperature also affects equipment stability: amplifiers, data acquisition systems, and uninterruptible power supplies (UPS) generate heat during operation, and insufficient cooling can lead to thermal noise, baseline drift, and reduced SNR. Maintaining precise temperature control helps minimize physiological variability and reduce equipment-related artifacts, increasing the reliability of recorded signals.

##### Humidity control

Relative humidity should be maintained between 40 and 60% to optimize electrode adhesion, ensure stable electrical conductivity, and preserve laboratory equipment (Zhu et al., 2020). Low humidity can cause electrode gel to dry too quickly, increasing impedance and degrading EEG signal quality. Excessive humidity can make conductive gel overly fluid, reduce participant comfort, and accelerate wear on electronic components. Stable humidity levels help preserve consistent data quality, maintain participant comfort, and prolong the lifespan of sensitive instrumentation.

##### Ventilation and air circulation

Adequate ventilation is necessary to prevent overheating from high-powered equipment while maintaining appropriate humidity levels. Poor airflow can create difficulties during extended recording sessions, particularly those lasting longer than 20 min. Insufficient ventilation increases perspiration, which alters electrode–scalp impedance and produces large voltage shifts known as cephalic skin potentials (Kappenman & Luck, 2010). These artifacts are especially problematic for high-impedance EEG systems, where sweat-induced impedance changes further weaken the scalp connection (Ledwidge et al., 2018). Ventilation systems must therefore provide sufficient airflow while operating quietly to avoid introducing acoustic noise into the recording environment. Maintaining controlled ventilation supports participant comfort, stabilizes equipment performance, and helps prevent data degradation.

##### Air quality and chemical emissions

Air quality is a critical factor in controlled laboratory settings, as elevated CO_2_ concentrations and volatile organic compounds (VOCs) can impair cognitive performance, reduce comfort, and affect sensor stability. CO_2_ levels above 1,000 ppm have been associated with reduced alertness, increased fatigue, and impaired decision-making (Allen et al., 2016). CO_2_ levels should be kept below 800 ppm to ensure adequate ventilation and fresh air exchange.

VOCs and formaldehyde must also be minimized to prevent health risks and avoid contamination of sensitive electronic components. VOC emissions originating from furniture, cleaning agents, and building materials are a major contributor to indoor air pollution (D’Amico et al., 2020; J. J. Zhang et al., 2022) and have been linked to headaches, dizziness, and respiratory irritation (Sundell, 2004).

Best practices for maintaining air quality include using low-emission construction materials, ensuring proper ventilation, and conducting periodic monitoring of VOCs and CO_2_. Implementing real-time air quality sensors helps maintain conditions within recommended thresholds, supports participant well-being, and minimizes potential interference with sensitive laboratory equipment.

#### Lighting

Proper lighting design is essential in multimodal neuroscience laboratories to minimize visual fatigue, ensure participant comfort, and prevent interference with neurophysiological recordings. Lighting conditions can influence cognitive performance, mental states, and signal quality, making adjustability, indirect illumination, and spectrum control important considerations (Liu et al., 2024; Lu et al., 2020; Tong et al., 2023). Natural light and windows should be avoided to maintain consistent and controlled experimental conditions.

Indirect lighting is recommended to reduce glare and visual strain for both participants and researchers (Lu et al., 2020). Neutral color temperatures around 4,000 K help create a comfortable and visually efficient environment and have been associated with improved reading performance and stabilized EEG activity (Lu et al., 2020). Illuminance levels should be maintained between 300 and 500 lx to provide adequate brightness without overstimulation or discomfort (C. Liu et al., 2024; Lu et al., 2020; Tong et al., 2023). Matte finishes and nonreflective surfaces further reduce glare and enhance visual comfort during tasks.

A configurable lighting system—such as a digital addressable lighting interface (DALI)—enables precise control over brightness, color temperatures, and lighting zones, accommodating a wide range of experimental demands. Automated protocols can adjust lighting based on time of day or experimental phases, ensuring consistent environmental conditions. For studies involving circadian rhythms, lighting systems should be capable of simulating day/night cycles, as research indicates that cool lighting during the day and warm lighting at night supports natural biological rhythms (Higuchi, 2024; Moore-Ede et al., 2023). In visual perception studies, controlling the spectral content of ambient light is crucial to prevent unintended interactions with visual stimuli and avoid variability across recording sessions.

Special considerations apply to fNIRS and other infrared-sensitive technologies. Natural light can introduce noise in the 700–900 nm wavelength range, which overlaps with the optical measurement band used in fNIRS, and should therefore be excluded. Infrared surveillance cameras must also be carefully managed, as their emissions may interfere with fNIRS sensors. Ensuring proper spectral control is thus critical for maintaining data quality when using infrared-based modalities.

#### Design strategy overview

Design decisions in multimodal neuroscience laboratories often involve selecting among several technically valid solutions that differ in cost, installation complexity, and flexibility for future upgrades. Table 2 summarizes these choices by grouping each design component into essential, recommended, optional, and future-proofing categories. Essential elements represent the minimum requirements for achieving stable, artifact-resistant neurophysiological recordings. Recommended components provide meaningful improvements in isolation, environmental regulation, and workflow efficiency. Optional elements offer further enhancements that may be useful when space, budget, or specific research objectives permit. Future-proofing components refer to design choices that are not required for current experimental needs but substantially reduce renovation costs or structural modifications when incorporating advanced modalities or stricter isolation requirements later on.

**Table 2:** Summary of core design components organized by essential, recommended, optional, and future-proofing categories

Category	Selected solution	Why?
**(a) Essential elements**		
Layout	Recording room with a dedicated control zone; enough space for efficient workflow	Ensures minimal controlled recording conditions with a reduction of researcher-participant interference
Acoustic treatment	Soundproofing walls with internal sound-absorbing panels	Ensures sufficient sound attenuation; avoids the structural demands of floating rooms
Electromagnetic shielding	Conductive paint shielding with grounding and shielded wired cabling	Offers baseline attenuation for common EMI sources; stabilizes the electrical environment; low cost; easy installation; supports incremental upgrades
Environmental controls	Stable temperature, humidity, and quiet ventilation, ensure fresh air exchange	Prevents signal drift, maintains electrode performance, and supports participant comfort and cognitive stability
Lighting	Indirect LED lighting (4000 K, 300–500 lx) with matte, non-reflective surfaces	Reduces glare and improves visual comfort while stabilizing neurophysiological recordings
**(b) Recommended elements**		
Layout	Physically separated two-room layout; no windows (wired audiovisual communication); buffer zones around the lab; enough space for hyperscanning	Improves participant isolation and structured cable routing; ensures hyperscanning capabilities
Acoustic treatment	Double-layer sound-proofed walls, floor, and ceiling with internal absorptive panels, full acoustic sealing, and a high-STC acoustic door	Enhances sound isolation and acoustic conditioning
Electromagnetic shielding	Conductive paint with multiple layers, dedicated grounding, and metallic shielding of ventilation ducts and transformers	Improves attenuation across broadband EMI sources and enhances SNR of recorded signals
Environmental controls	Low-noise HVAC with controlled airflow, humidity stabilization, and low-emission interior materials	Improves physiological stability, reduces mechanical noise, and minimizes air-quality fluctuations that can affect recordings
Lighting	DC-powered adjustable LED lighting with spectral control and independently addressable zones; no natural light exposure	Supports diverse experimental paradigms and improves reproducibility; no interference with fNIRS
**(c) Optional elements**		
Layout	Addition of a relaxation area or room for participants	Ensures a reduction of the participant's cognitive load before the experiment, but requires more space
Acoustic treatment	Floating floors and advanced diffusion devices, such as Schroeder diffusers	Offer additional vibration control, but require high cost and structural modification
Electromagnetic shielding	Metallic mesh in the walls, ceiling, and floor	Provides very high isolation but requires invasive installation, higher cost, and reduced flexibility for future room modifications
Environmental controls	Real-time CO_2_ and VOC monitoring systems with automated ventilation adjustment	Enhances environmental control, but is not necessary for standard multimodal studies
Lighting	Circadian-capable or fully dynamic spectral lighting systems for sleep or chronobiology studies	Provides advanced environmental control but exceeds the requirements of standard cognitive or multimodal experiments
**(d) Future-proofing elements**		
Layout	Additional space, rooms, and infrastructure to support new recording technologies or multiple simultaneous experiments	Reduces the need for structural changes when adding new equipment (e.g., TMS, MEG, or fMRI) or expanding experimental capacity
Acoustic treatment	Structural allowance for future floating-room upgrades	Reduces the need for major reconstruction if stricter acoustic isolation becomes necessary
Electromagnetic shielding	Base shielding infrastructure that can be upgraded with additional copper mesh or magnetic shielding layers as required for advanced modalities	Reduces the need for major reconstruction when integrating more EMI-sensitive technologies—such as MEG, TMS systems, or fMRI—or when expanding the recording setup
Environmental controls	Infrastructure allowing independent HVAC lines and upgraded filtration or air-monitoring systems	Reduces the need for structural changes if stricter thermal, airflow or air-quality control becomes necessary for advanced paradigms
Lighting	LED drivers with digital control interfaces supporting future integration of advanced spectral systems or modality-specific lighting	Allows adding more sophisticated or modality-compatible lighting without redesigning the electrical infrastructure

Essential elements constitute the minimum requirements for stable multimodal recordings; recommended elements improve isolation and system performance; optional elements provide situational enhancements; and future-proofing elements facilitate later integration of advanced modalities without major reconstruction.

This structure clarifies the rationale behind key design decisions. For example, conductive paint is categorized as essential because it offers adequate EMI attenuation at low cost and with minimal installation burden, whereas copper mesh is listed as optional due to its higher shielding performance but greater structural demands. Similarly, standard soundproofing materials provide sufficient acoustic isolation for most multimodal laboratories, while floating floors or advanced diffusers are reserved for settings that require more stringent vibration control or acoustic scattering management. Future-proofing elements—such as base EMI shielding capable of supporting additional layers or HVAC systems sized for higher filtration loads—ensure that the laboratory can later accommodate technologies such as TMS-, MEG-, or fMRI-compatible equipment without major structural reconstruction.

### Acquiring and integrating research equipment

The selection of equipment for measuring and analyzing brain activity in a multimodal neuroscience laboratory is complex and highly dependent on experimental requirements. This section provides an overview of essential laboratory components, including EEG, fNIRS, eye-tracking systems, and peripheral biosensors, and addresses considerations related to software, multimodal integration, and data management.

#### General lab equipment

In experimental psychological and cognitive neuroscience research, precise stimulus delivery and accurate response measurement are fundamental for producing valid and reproducible findings. A well-equipped laboratory must therefore include devices capable of presenting stimuli with controlled timing and capturing behavioral responses with high temporal fidelity.

##### Stimulus presentation equipment

Stimulus presentation tools must ensure accurate delivery across visual, auditory, and multisensory modalities. High refresh rate monitors are critical in visual paradigms, particularly those involving motion perception or rapid visual processing. Increasing refresh rates has been shown to increase the intensity of motion-related visual evoked potentials (Han et al., 2022). To minimize latency and motion blur, monitors with a minimum refresh rate of 120 Hz are recommended, with 240 Hz or higher preferred for tasks requiring precise motion representation (Han et al., 2022). Reading and linguistic experiments may also require high refresh rates to support precise temporal alignment of character, syllable, word, phrase, or sentence-level responses.

For auditory stimulation, systems with flat frequency responses are required to avoid distortions that may influence perception or cognitive processing (Scharf, 1998). Because the recording environment shapes the radiated acoustic field according to its modal resonances, room characterization and calibration of audio devices are essential for achieving reproducible acoustic conditions. In EEG studies, in-ear headphones are generally recommended, as over-ear models can interfere with electrode caps and introduce mechanical artifacts (Light et al., 2010; Vakhrushev & Pooresmaeili, 2024). Over-ear designs may be used in studies not involving EEG or fNIRS. Regardless of form factor, consumer and studio headphones typically exhibit nonlinear frequency responses; flat-response models are therefore preferred to minimize spectral distortions.

Closed-back or active noise-cancelling (ANC) designs help reduce external distractions, particularly in acoustically suboptimal environments. Loudspeakers may be used when spatial hearing or naturalistic listening environments are required, but must be equalized to achieve a flat frequency response and to compensate for room-induced distortions (Brungart & Rabinowitz, 1999). This is especially important in research involving sound localization, auditory scene analysis, or speech comprehension under dynamic conditions.

Multimodal experiments may incorporate additional sensory modalities—such as tactile, electrical, or olfactory stimulation—which require precise control and synchronization. These methods fall outside the scope of the present guidelines due to their specialized nature, but laboratories adopting such modalities should ensure compatibility with neurophysiological recordings and validate timing accuracy for the specific application.

##### Response recording equipment

Accurate response measurement is essential for linking behavioral and neurophysiological data. Standard computer keyboards introduce variable latency and are unsuitable for precise reaction-time measurements. Dedicated response devices (e.g., USB response pads by Black Box Toolkit) provide sub-millisecond accuracy and interface directly with stimulus software to ensure consistent timing (Garaizar et al., 2014). Device selection should be matched to task demands: pointing devices or computer mice are preferred for tasks requiring fine motor control or gaze-contingent interactions, while mechanical keyboards with low debounce times can provide more reliable timing than standard membrane keyboards when button responses are required (Plant & Turner, 2009).

Beyond manual response tools, cameras are widely used for behavioral analysis and participant monitoring. High-speed cameras operating at several hundred frames per second offer detailed analysis of movement, facial expressions, and nonverbal behavior (Ekman & Friesen, 2019). Motion-capture and biomechanics systems provide even higher spatial and temporal resolution and are suitable for research on motor control, rehabilitation, and sensorimotor integration.

#### Research equipment

A multimodal neuroscience laboratory requires specialized equipment capable of capturing and integrating neurophysiological and behavioral signals. Although each modality has distinct technical specifications, several overarching considerations guide system selection, including data quality, multimodal compatibility, synchronization requirements, portability, and interoperability with third-party software. Tables S1–8 in Supplementary Materials summarize commercially available EEG, fNIRS, eye-tracking, and peripheral biosensor systems, outlining their specifications and suitability for diverse research needs.

##### EEG systems

Electroencephalography (EEG) remains a central tool in cognitive neuroscience due to its high temporal resolution and direct measurement of neural oscillations. Selecting an EEG system requires careful evaluation of amplifier performance, electrode type and density, impedance characteristics, and spatial/temporal resolution, as these factors directly influence signal quality, setup efficiency, and experimental flexibility.

High-quality amplifiers are essential for minimizing noise and preserving signal integrity. Amplifiers with high input impedance (> 1 GΩ) reduce signal attenuation and vulnerability to external contamination, while a CMRR above 100 dB helps suppress environmental interference. Some systems employ active electrodes that amplify the signal at the electrode site, improving SNR and reducing preparation time. Active shielding mechanisms may further enhance noise suppression under challenging environmental conditions.

Electrode type also influences data quality and usability. Dry electrodes facilitate rapid setup but produce higher impedance and are more susceptible to motion artifacts (Q. Liu et al., 2023). Semi-dry or hybrid electrodes, which use small volumes of saline solutions, allow faster preparation but may require refreshment during recordings, making them suitable for time-sensitive protocols (Q. Liu et al., 2023). Wet electrodes, particularly sintered silver/silver chloride (Ag/AgCl), offer the most stable and highest-fidelity signals but require conductive gel and longer preparation and cleanup times.^2^

Electrode density determines spatial resolution and preparation demands. High-density EEG systems (128–256 channels) improve source localization at the expense of longer setup and more complex processing pipelines. Low-density systems (9–32 channels) provide increased stability and lower impedance, yielding robust data with fewer artifacts but reduced spatial precision. As noted in prior work, increased density does not always guarantee superior data quality, particularly when considering artifacts such as cephalic skin potentials (Luck, 2014).

Impedance management is another critical factor. Low-impedance electrodes require scalp abrasion, which may reduce comfort but provides superior stability. High-impedance systems simplify preparation but are more sensitive to fluctuations in skin conductance (Ledwidge et al., 2018). EEG temporal resolution depends on sampling rate and system bandwidth; most cognitive neuroscience applications require bandwidths of 120–150 Hz and sampling rates between 240 and 300 Hz (Ray et al., 2008; Valderrama et al., 2012), while studies targeting high-frequency activity may require rates above 1,000 Hz.

The choice between wired and wireless systems depends on experimental constraints. Wired systems offer maximal data fidelity and minimal susceptibility to EMI, making them preferable for high-precision research. Wireless systems increase participant mobility but introduce latency and potential signal degradation, making them more suitable for naturalistic or mobile applications. Many modern EEG systems support integration with other modalities (e.g., fNIRS), enabling synchronized multimodal acquisitions (see the Multimodal Integration section).

##### fNIRS systems

fNIRS measures cortical hemodynamic activity and complements EEG by providing insights into neurovascular dynamics. Optode placement, signal quality, system resolution, and multimodal compatibility are key considerations in selecting an fNIRS system.

Optode configuration strongly influences data quality and cortical coverage. Flexible and adjustable optode placement allows researchers to accommodate different head sizes and target specific cortical regions. Automated optode array design has gained interest as a means to streamline setup and improve standardization (Zimeo Morais et al., 2018). fNIRS systems typically employ either modular optodes—which maximize flexibility but are more prone to movement artifacts—or embedded optodes, which sacrifice flexibility in exchange for improved SNR due to integrated shielding.

The number of channels depends on the arrangement of light sources and detectors, and only detector-source pairs positioned at appropriate distances yield usable data. SNR declines sharply when distances exceed optimal thresholds. An approximate 3-cm source–detector spacing provides ~ 1.5 cm cortical penetration and represents a standard compromise between sensitivity and SNR. Short-separation channels (~ 8 mm) should be included to capture superficial hemodynamic and improve artifact correction (Brigadoi & Cooper, 2015).

Signal quality depends on source–detector distance, light source type, sampling rate, and environmental conditions (Zimeo Morais et al., 2018). Light-emitting diode (LED)-based systems are common in portable devices due to low power consumption, whereas laser-based systems offer higher power and deeper penetration, making them preferable for high-precision research. Although spatial resolution remains lower than fMRI (Sakai, 2022), advances in high-density fNIRS (HD-fNIRS) have significantly improved spatial precision.^3^ Unlike fMRI, fNIRS maintains advantages in portability and motion tolerance, enabling naturalistic studies involving speech, social interaction, and motor behavior.

Typical sampling rates range from 10 to 100 Hz, although some systems achieve 240 Hz for applications requiring rapid hemodynamic measurements. While fNIRS does not match the millisecond precision of EEG, it balances moderate temporal resolution and high ecological flexibility.

Environmental considerations are critical. Since fNIRS operates in the near-infrared spectrum, interference from external infrared sources (e.g., eye-tracking devices, thermal or biomechanical motion capture cameras) may introduce flickering artifacts or spectral contamination if wavelengths overlap. Mitigation strategies include synchronized pulsing, filtering, or shielding. Ambient lighting also affects optical noise. Overhead (cenital) lighting can create unwanted reflections or fluctuating light intensity. Indirect LED lighting or DALI-controlled lighting reduces these artifacts and maintains comfortable visibility for participants and researchers.

##### Eye-tracking systems

Eye-tracking technology provides essential measures of visual attention, perception, and decision-making (Skaramagkas et al., 2023). System selection should consider accuracy, precision, sampling rate, calibration reliability, latency, and integration with other modalities.

Modern systems typically achieve accuracies between 0.5° and 1° of visual angle, sufficient for most cognitive and perceptual research. Precision—reflecting the stability of measurements—is enhanced by filtering algorithms that reduce head movement and environmental artifacts. Sampling rates of at least 200 Hz are recommended for general cognitive studies (Andersson et al., 2010), while frequencies exceeding 1,000 Hz are required for microsaccade detection or detailed ocular dynamics as in linguistics studies (Angelopoulos et al., 2021).

System configuration depends on research goals. Remote eye-trackers provide high accuracy with minimal participant burden and are suited to stationary setups. Wearable systems enable mobile studies—including virtual reality (VR), augmented reality (AR), and real-world exploration—while head-supported systems offer maximal stability at the cost of reduced comfort during long sessions. Calibration remains essential; modern systems incorporate adaptive recalibration to maintain accuracy in the presence of minor head movements (Gunawardena et al., 2023; Hassoumi et al., 2019).

Latency is another important factor, particularly in real-time or interactive paradigms (e.g., VR-based studies). Most modern systems achieve delays between 45 and 81 ms, whereas specialized high-end devices reach latencies as low as 3 ms, supporting VR and closed-loop real-time applications (Stein et al., 2021).

##### Peripheral biosensors

Peripheral biosensors capture autonomic and motor system activity, complementing EEG and fNIRS measurements. They support analyses of heart rate variability (HRV), skin conductance, muscle activity, respiratory patterns, and movement dynamics. System selection depends on research goals, required biometrics, participant comfort, and synchronization capabilities.

Modularity is an important consideration when selecting peripheral biosensors. Many EEG systems offer auxiliary inputs for adding physiological sensors as needed. For example, studies focused on the regulation of the autonomic nervous system may require an ECG for HRV measurements or GSR sensors for assessing emotional and stress-related responses. Conversely, motor control research may require electromyography (EMG) sensors to track muscle activation and coordination patterns. Different physiological processes exhibit different temporal dynamics; thus, sampling rates must match signal characteristics. High-speed physiological events (e.g., cardiac activity, muscle potentials, saccadic eye movements) typically require sampling rates above 250 Hz (Shaffer & Ginsberg, 2017). Slower signals, such as GSR or accelerometry, tolerate lower sampling frequencies without compromising accuracy.

For long-duration or naturalistic studies, wearability and comfort are key to minimizing behavioral disruption. Traditional wired systems provide superior fidelity and are well suited for controlled environments, but advances in wearable technology have produced wireless systems capable of high sampling rates and stable performance (Xiang et al., 2020).

Commercial biosensor systems vary in channel count, signal quality, and integration options. Some include built-in sensors; others allow modular configurations. Signal quality may be enhanced through active shielding and high sampling rates (up to 400 kHz in advanced models). Many modern systems support real-time processing, enabling closed-loop applications and online analysis.

#### Multimodal integration and interferences

Multimodal integration leverages the complementary strengths of different recording techniques to provide a more comprehensive understanding of brain function and physiological responses. EEG offers millisecond temporal precision, while fNIRS contributes spatially resolved hemodynamic information, making the combination particularly powerful. However, integrating multiple modalities introduces technical and logistical challenges that must be managed carefully to ensure high-quality data collection. Simultaneous operation of multiple systems can create cross-interference and signal contamination (Table 3), reducing data reliability and necessitating targeted mitigation strategies.

**Table 3:** Summary of neuroscience recording techniques, sensor types, and applications

Technique	Sensor type	Technology	Temporal resolution	Spatial resolution	Portability	Example applications
EEG	Electrodes	Electrical signals	High (milliseconds)	Low (cm range)	High (wearable & lab)	Brain activity monitoring, BCI, cognitive studies
fNIRS	Optodes	Infrared	Low (seconds)	High (mm range)	Moderate (wearable & lab)	Hemodynamic responses, cognitive studies
Eye-tracking	Cameras	Infrared	High (milliseconds)	High (sub-mm to mm)	High (wearable & lab)	Visual attention, reading studies, user experience (UX)
Peripheral biosensors	Electrodes (ECG, EMG, GSR, etc.)	Electrical signals	High (milliseconds)	Moderate (cm range)	High (wearable)	Heart rate, stress studies, and muscle activity
Hyperscanning	Multimodal (EEG, fNIRS, eye-tracking, etc.)	Multimodal (all above)	Varies by modality	Varies by modality (mm–cm range)	Moderate to low	Multi-brain interactions, social cognition

Overview of key neuroscience recording techniques, including sensor types, underlying technology, temporal and spatial resolution, portability, and example applications. The table highlights the strengths and limitations of each modality, emphasizing their suitability for different research contexts, such as cognitive studies, brain stimulation, social cognition, and physiological monitoring. Note that modern EEG systems incorporating active electrodes are generally less susceptible to electromagnetic interference than passive systems, due to preamplification at the electrode site.

##### Multimodal interferences

A primary challenge in multimodal setups is sensor placement competition. Accurate positioning of EEG electrodes, fNIRS optodes, and peripheral biosensors is essential to avoid channel overlap, preserve cortical coverage, and maintain signal integrity. Modern hybrid EEG-fNIRS caps can help streamline sensor placement and reduce interference (see Tables S2 and S4 in Supplementary Materials). Systems offering auxiliary channels—typically EEG—provide flexibility for incorporating additional sensors such as GSR, ECG, and EMG. However, because EEG and most peripheral biosensors rely on electrodes, they are susceptible to crosstalk when placed too closely together. Capacitive coupling—where electrical signals from one sensor interfere with another—can degrade data quality. This can be mitigated using shielded cables, shielded power supplies, and active electrodes, alongside maintaining low electrode impedance.

Technological interference across modalities also presents challenges. Both eye-tracking systems and fNIRS devices operate in the infrared spectrum and can disrupt one another’s measurements. Infrared emission from fNIRS sources may interfere with eye-tracking cameras, while infrared-based eye trackers may introduce noise to fNIRS detectors. Verifying wavelength compatibility between systems is necessary to avoid these conflicts. Optode placement should avoid the frontal region in setups involving infrared eye-tracking, reducing the risk of cross-contamination. Black or nonreflective optode caps can further minimize infrared reflections and improve data quality of both modalities.

Temporal resolution differences must also be considered when designing multimodal protocols. EEG captures rapid electrical activity on the millisecond scale, whereas fNIRS measures hemodynamic responses that evolve over several seconds. EEG-fNIRS paradigms, therefore, require tasks that accommodate the slower temporal dynamics of hemodynamic responses. By contrast, EEG and eye-tracking share high temporal resolution, making them well suited for paradigms requiring precise alignment of neural and oculomotor events. Failure to account for these timing differences can lead to misaligned datasets and incorrect temporal interpretations.

##### Multimodal integration

Accurate synchronization is critical for all multimodal recordings, as even minor timing discrepancies can distort temporal relationships between physiological signals. Synchronization is required not only across modalities within a single participant but also across participants in hyperscanning studies. Some EEG and fNIRS systems provide native synchronization when purchased from the same manufacturer, but integrating hardware from different manufacturers often requires additional synchronization approaches due to discrepancies in sampling rates and internal clocks (see Supplementary Materials for a summary of synchronization capabilities of commercially available EEG, fNIRS, eye-tracking, and biosensor systems).

Hardware-based synchronization—using transistor–transistor logic (TTL) triggers, photosensors detecting screen luminance changes, or dedicated synchronization devices such as the TriggerBox (Brain Products)—provides the most reliable alignment by minimizing latency and jitter. When hardware synchronization is not possible, software-based solutions such as lab streaming layer (LSL) or TCP/IP timestamping can be used for post hoc alignment. These methods help maintain temporal precision across diverse multimodal systems and ensure accurate interpretation of relationships between signals.

##### Cable management and data transmission integrity

Stable and interference-free data transmission is essential in multimodal setups, especially when multiple devices operate simultaneously or when recording and control rooms are separated. Proper cable management reduces electromagnetic noise, prevents physical strain on connectors, and preserves signal integrity.

Wall-mounted connection panels in both recording and control rooms help organize cables and minimize clutter. Shielded conduits and properly grounded power distribution systems reduce EMI contamination. Long-distance data transmission—such as USB 3.0 runs longer than 5 m—requires high-quality active extension cables to maintain signal integrity. USB extenders and daisy-chained adapters should be avoided, as additional connection points increase the risk of degradation and instability (insertion losses).

##### Software considerations

Software is fundamental for managing, synchronizing, and analyzing multimodal datasets. Effective software solutions must support compatibility across recording systems, particularly in hyperscanning studies or when hardware from different manufacturers is used. Many EEG systems integrate directly with fNIRS, and both TTL triggers and LSL facilitate cross-system synchronization.

Compatibility across operating systems (Windows, macOS, Linux), and availability of software development kits (SDKs) and application programming interfaces (APIs) further enhance flexibility, enabling custom data acquisition pipelines and integration with third-party applications. Post-processing tools remain essential for handling large multimodal datasets, supporting artifact correction, synchronization, and statistical analysis. Table S9 in the Supplementary Materials summarizes widely used software solutions and their capabilities.

##### Data management and compliance

Multimodal experiments generate substantial data volume, requiring robust storage and backup strategies to ensure long-term integrity, accessibility, and security. Cloud platforms, local hard drives, and network-attached storage (NAS) systems should be used in combination, with automated backups providing redundancy and protection against data loss.

Data privacy is critical when handling sensitive behavioral and physiological information. Compliance with regulations such as the General Data Protection Regulation (GDPR, 2016) in Europe—and equivalent national laws elsewhere—requires strict control over data access, encryption protocols, and anonymization procedures. Participant data must be stored securely, processed transparently, and retained only as long as necessary for the research objectives.

Consent forms and related documentation must also be stored securely. Best practices recommend scanning and encrypting digital copies for archive while keeping physical originals in locked storage. When working with minors or vulnerable populations, researchers must obtain parental or guardian consent and comply with appropriate child data protection regulations.

To support long-term usability and reproducibility, laboratories should adopt standardized metadata frameworks such as Dublin Core or the brain imaging data structure (BIDS) for neuroimaging data. Standardization in data handling and documentation reduces methodological variability, facilitates cross-study comparisons, and promotes reproducibility and transparency in cognitive neuroscience (Nebe et al., 2023).

## Practical application: INAB Laboratory

The following section describes the practical implementation of the design principles outlined above in a multimodal neuroscience laboratory dedicated to experimental psychology and cognitive neuroscience research. We detail the laboratory’s physical layout, selected technologies and equipment, and the environmental measures used to ensure optimal performance and data quality. We also report key results from acoustic and electromagnetic interference assessments, together with the deployment of lighting scenarios. By linking the general guidelines to a concrete implementation, this section illustrates both the outcomes and the practical challenges encountered during the setup process.

### Laboratory design and infrastructure modifications

#### Location and layout

The laboratory is located in the Norrsken building on Barceloneta Beach (Barcelona, Spain) in an area isolated from high-traffic zones. The building is equipped with advanced HVAC systems designed to maintain stable temperature and humidity levels while minimizing acoustic and vibrational noise. The HVAC installation includes low-noise air handling units with high-efficiency filtration and independent temperature control for each room, ensuring appropriate conditions for participants, researchers, and sensitive equipment.

The laboratory area is structurally isolated from the main working zones and includes buffer spaces and a dedicated cleaning area. The room is not directly connected to the building facade, which reduces exposure to external vibroacoustic and electromagnetic noise. A dedicated waiting area was established outside the lab to maximize participant comfort and data quality by allowing participants to relax and acclimate before experiments. This space supports a calm and focused state, helping to stabilize physiology before recording. Ergonomic chairs and tables made from natural materials were selected to enhance comfort, and walls were painted a neutral tone (NCS 1002-Y50R), free of logos, pictures, or windows, to minimize distraction.

**Fig. 1:**
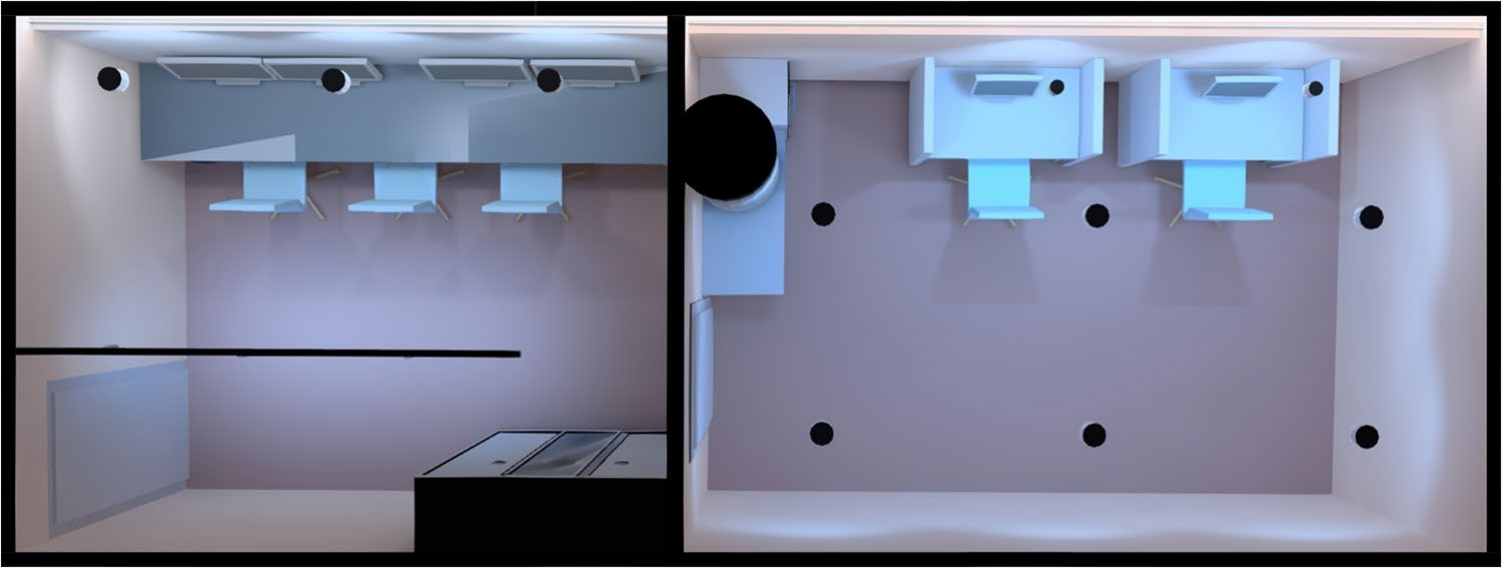
Laboratory two-room layout of the preliminary design. The control room (left) and the recording room (right) are shown, along with key functional areas: the participant working stations, the researcher preparation stations, the storage for equipment, the computers for processing and recording the data, and the lighting system (designed with the digital addressable lighting interface (DALI) system, represented by the small black dots on the ceiling)

**Fig. 2:**
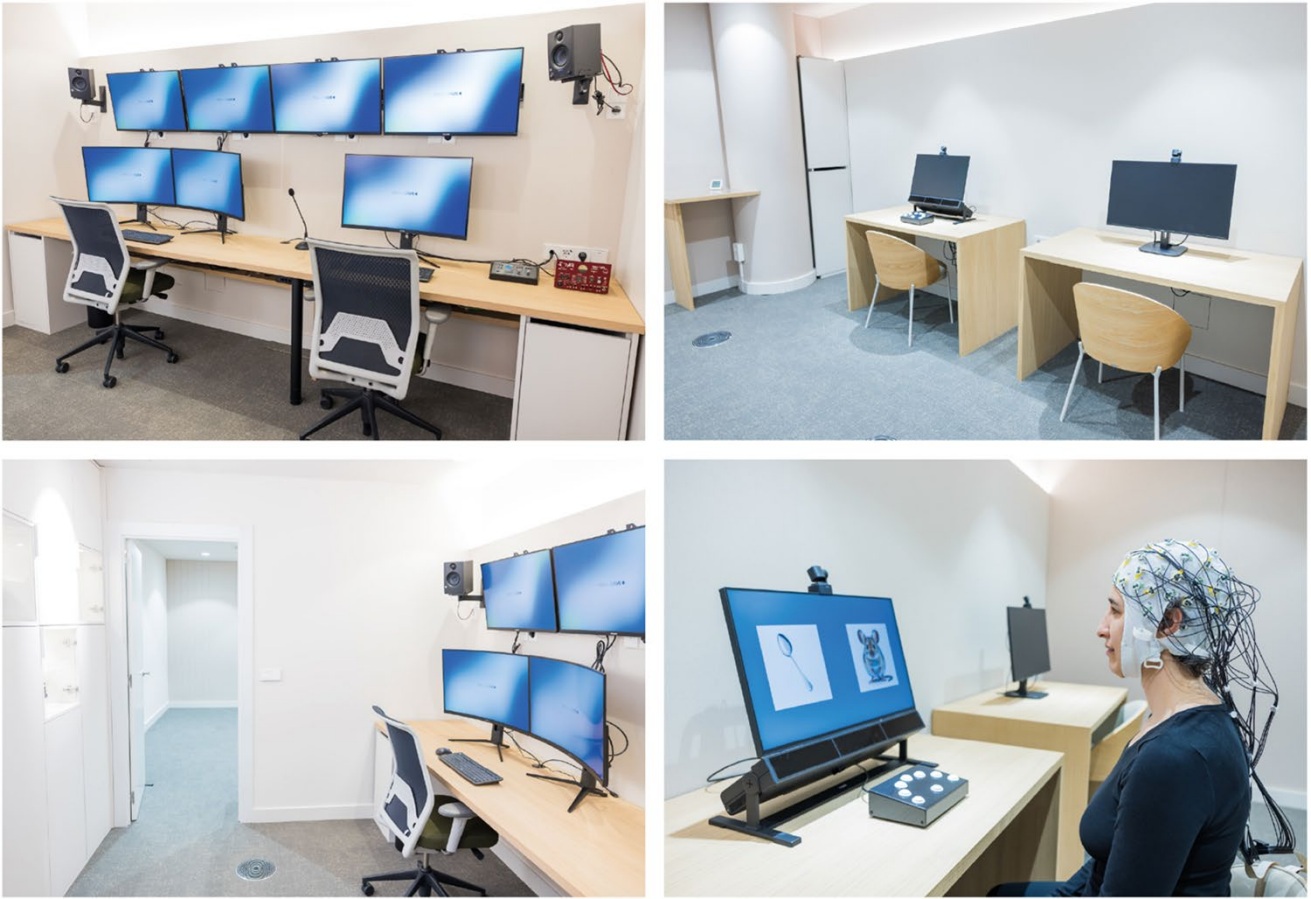
Portfolio of pictures of the Instituto de Neurociencia Avanzada de Barcelona (INAB) laboratory. The control room (top left), the recording room (top right), and the hallway connecting the two rooms (bottom left) are depicted. An experimental session in progress is also shown (bottom right), with a participant wearing an EEG cap while doing a visual perception task

The laboratory volume measures 2.25 m in height, 8.77 m in depth, and 3.14 m in width, and is divided into two areas: a control room and a recording room (Figs. 1 and 2). This two-room configuration was chosen to balance isolation and budget constraints.^4^ Communication between rooms is provided via a microphone-speaker system, enabling clear interaction without compromising the recording environment (see “General lab equipment”). The recording room includes two dedicated experimental positions, allowing multiple participants to be tested in parallel and supporting hyperscanning protocols. Custom worktables and cabinets were designed to match the laboratory’s specific requirements, facilitating equipment storage and access. A mobile trolley for recording equipment is used to streamline setup at the participant’s position.

The control room includes two spacious workstations dedicated to data recording and processing. Multiple monitors allow visualization of experiment protocols, camera feeds, and biosignal displays, enabling real-time monitoring and adaptation during data acquisition. Together, these design elements provide an efficient, adaptable, and participant-friendly research environment that supports high data integrity and robust experimental control.

#### Acoustic treatment

The laboratory was configured as a speech-optimized, acoustically controlled space to improve sound clarity and reduce external noise interference. Acoustic properties were first assessed using EASE (Enhanced Acoustic Simulator for Engineers, version 4.3), simulating the room under two conditions: before and after acoustic panel installation. The simulation domain was discretized using a 1-cm grid for high spatial resolution. Sound absorption data were obtained from published sources (Carrión Isbert, 1998; Celenit, 2025; Summers, 2008). Key simulated parameters included reverberation time (RT), clarity index (C50), speech transmission index (STI), and consonant articulation index (AICons).

Empirical reverberation measurements were taken at six positions within the room before and after panel installation, following ISO 3382–1 (ISO 3382–1, 2009) and ASTM E2235 (ASTM E2235-04, 2020). Measurements were made using a sound level meter (Rion NA-28) and covered frequencies from 20 Hz to 20 kHz under controlled conditions (22 °C, 35% humidity).

Fiberglass soundproofing was installed inside the walls to improve isolation, and 9 mm acoustic panels made from biocompatible, sustainable materials (EcoCero, V-cut) were mounted to enhance acoustic comfort and reduce external noise transmission (del Rosario-Gilabert et al., 2024). The noise reduction coefficient (NRC) of these panels is reported in Table S10 (Supplementary Materials).

Simulations before and after panel installation indicated substantial improvements (Table 4). The C50 increased from − 2.3 dB to 3.5 dB, reflecting better control over early reflections and improved sound clarity. STI increased from 0.55 to 0.7, indicating enhanced speech intelligibility, which is important for experiment instructions and communications. Simulated RT decreased from 1.41 s to 0.56 s, showing a strong reduction in the reverberant energy, and AICons improved from 8.9% to 3.9%, indicating better consonant articulation and overall speech intelligibility.

**Table 4:** Simulated improvements in acoustic comfort within the recording room

Parameter	Without acoustic treatment	Rating	After acoustic treatment	Rating
C50 [in dB]	− 2.3	Poor	3.5	Good
STI	0.55	Acceptable	0.7	Good
%AlCons	8.9	Acceptable	3.9	Good
RT [in s]	1.41	Good	0.56	Optimal

Comparison of acoustic simulated parameters at 1 kHz, including reverberation time (RT), clarity index (C50), speech transmission index (STI), and articulation index of consonants (AICons) before and after acoustic treatment. Ratings are based on UNE-EN IEC 60268–16:2020 (2021) standards, demonstrating enhanced speech clarity, reverberation control, and overall acoustic comfort within the laboratory.

**Fig. 3:**
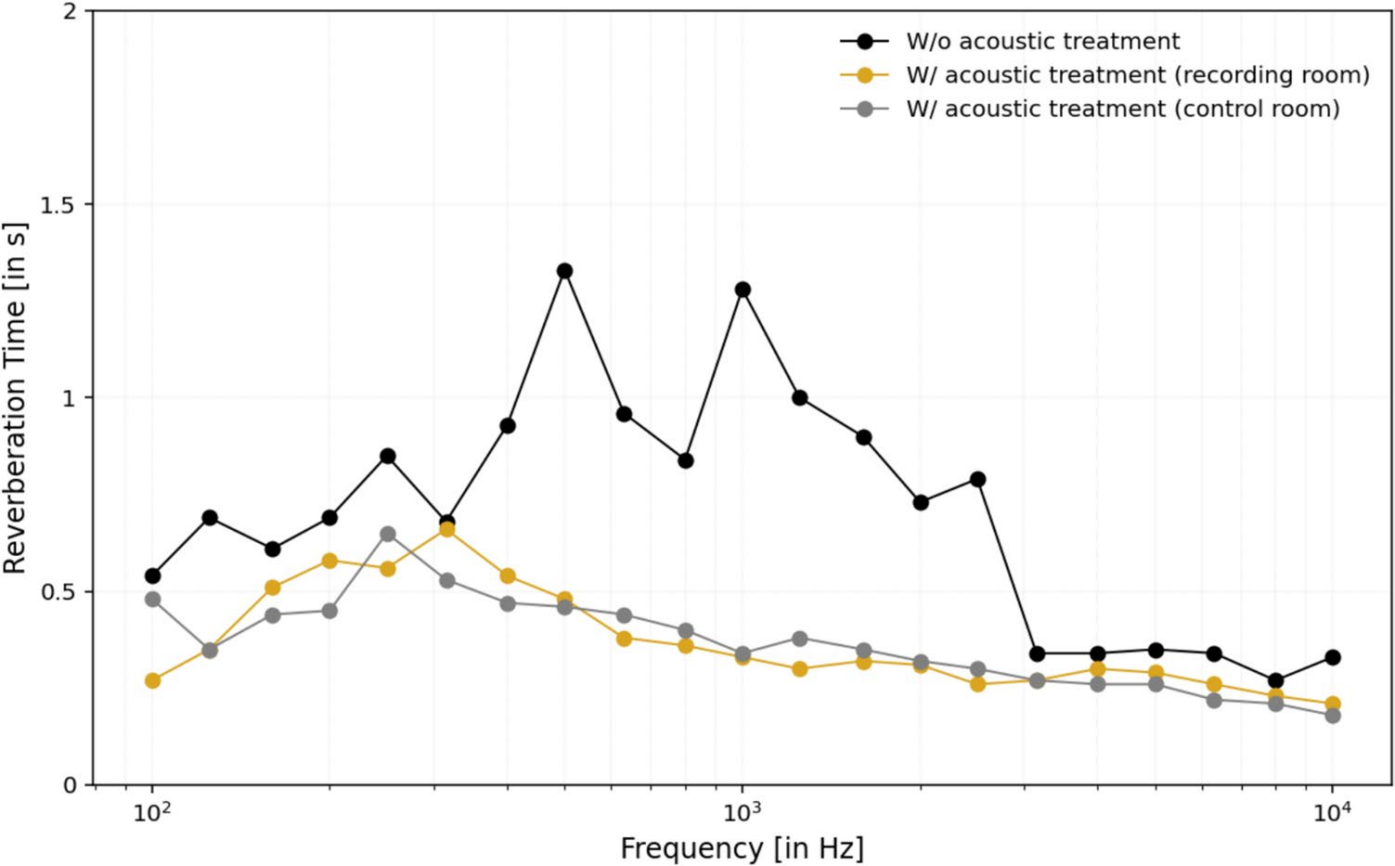
Experimental reverberation time (RT) measurements across different frequencies under three conditions: without acoustic treatment (blue circles), with acoustic treatment in the recording room (orange squares), and with acoustic treatment in the control room (green triangles). The untreated room shows significantly higher RT values, particularly in the mid-frequency range (~ 500 Hz to 2 kHz). Acoustic treatment effectively reduces reverberation, improving the sound field in both treated rooms and enhancing overall sound clarity and control

Measured RT values across 100 Hz to 10 kHz (Fig. 3) showed that, prior to treatment, RT exceeded 1 s at mid-frequencies. After treatment, RT was reduced to below 0.6 s across most of the spectrum, with particularly strong improvements at mid and high frequencies where absorptive materials are most effective. Differences between the recording and control rooms were small and likely to reflect variations in treatment distribution or geometry. Background noise equivalent level was 36.6 dBA (1 min), consistent with a quiet laboratory environment.

#### Electromagnetic shielding

A comprehensive EMI analysis was carried out using four specialized instruments in two measurement phases with different objectives.

In the first phase, prior to laboratory installation, the goal was to characterize the existing electromagnetic environment. Low-frequency measurements (40 Hz – 1 MHz) were obtained with an AARONIA NF-5035 spectrum analyzer, and high-frequency measurements (700 MHz – 6 GHz) with an AARONIA SPECTRAN HF-60105 V4 and an AARONIA OMNILOG-70600 omnidirectional antenna. Measurements were taken at two locations, 1.1 m above the floor, over 3-min intervals to assess EMI levels from existing electrical infrastructure. This approach yielded frequency-resolved root mean square (RMS) data to identify potential sources and verify compliance with exposure limits.

In the second phase, after laboratory installation and shielding, measurements focused on verifying shielding effectiveness in the control and recording rooms. Broadband electromagnetic field (EMF) meters from Gigahertz Solutions were used: the NFA1000 for low-frequency electric and magnetic fields (5 Hz – 1 MHz) and the HF59B for high-frequency radiation (27 MHz – 2.7 GHz), combined with a UBB27 omnidirectional antenna. Measurements were collected on three separate days at three locations per room (1.1 m height, 30 s per location) and focused on peak field values. These devices are optimized for practical field assessments and rapid detection of local maxima.

Absolute values from the two measurement phases are not directly comparable because the first phase used RMS-based spectral analysis, and the second used broadband peak detection. However, each method was appropriate for its intended purpose: environmental characterization in the first phase and shielding validation in the second. All measurements were evaluated relative to European and local regulations, including International Commission on Non-Ionizing Radiation Protection (ICNIRP) guidelines (ICNIRP, 1998) and Spanish Real Decreto 1066/2001 (1066/2001, 2001).

Table 5 summarizes results for electric field strength, magnetic flux density, and power density. Before the laboratory setup, a peak electric field of 15.3 V/m was detected at 132.6 kHz (Fig. S1 in Supplementary Materials), well below the 87 V/m reference level specified by ICNIRP (ICNIRP, 1998) for this range listed in Table 5. Magnetic flux density at 50 Hz was 0.023 µT, far below the public exposure limit of 200 µT. Maximum power density between 700 MHz and 6 GHz was 5.73 µW/m^2^, well under the 10 W/m^2^ ICNIRP limit. AARONIA reports measurement uncertainties of ± 3% for low-frequency fields and ± 1 dB for high-frequency fields.

**Table 5:** Electromagnetic field (EMF) measurements and regulatory limits before (**a**) and after (**b**) the laboratory setup

**EMF measurements**	**Recorded frequency range**	**Maximum RMS value**	**Frequency of maximum value**	**Regulatory limit (ICNIRP)**
**(a) Measurements before the laboratory setup (AARONIA equipment):** Maximum RMS values recorded using spectrum analyzers				
Electric field (E)	40 Hz–1 MHz	15.3 ± 0.46 V/m	132.6 kHz	87 V/m
Magnetic flux density (B)	40 Hz–1 MHz	0.023 ± 0.001 µT	48.8 Hz	200 µT
Power density (S)	700 MHz–6 GHz	5.73 ± 1.48 µW/m^2^	Filtered from 2.5 to 6 GHz	10 W/m^2^
**EMF measurements**	**Recorded frequency range**	**Peak value**	**Frequency of maximum value**	**Regulatory limit (ICNIRP)**
**(b) Measurements after the laboratory setup (Gigahertz Solutions equipment):** Maximum peak values recorded using broadband EMF meters				
Electric field (E)	5 Hz–1 MHz	Control room: 12.9 ± 0.7 V/m Recording room: 0.4 ± 0.07 V/m	50 Hz	87 V/m
Magnetic flux density (B)	5 Hz–1 MHz	Control room: 0.144 ± 0.008 µT Recording room: 0.059 ± 0.003 µT	50 Hz	200 µT
Power density (S)	27 MHz–2.7 GHz	Control room: 27.6 ± 11.3 mW/m^2^ Recording room: 0.714 ± 0.292 mW/m^2^	Filtered from 27 MHz to 2.7 GHz	10 W/m^2^

Summary of recorded EMF values, including electric field (E), magnetic flux density (B), and power density (S), measured across different frequency ranges. The table presents maximum recorded values, their corresponding frequency of occurrence, and the International Commission on Non-Ionizing Radiation Protection (ICNIRP) regulatory limits for comparison.

After laboratory installation and shielding, peak electric field strength reached 12.9 V/m at 50 Hz in the control room, but was reduced to 0.4 V/m at 50 Hz in the recording room. Magnetic flux density was 0.144 µT at 50 Hz in the control room and 0.059 µT at 50 Hz in the recording room. For high-frequency radiation, peak power density was 27.6 mW/m^2^ in the control room and 0.714 mW/m^2^ in the recording room. These measurements show that electric and high-frequency fields in the recording room are more than 30 times lower than in the control room, and magnetic fields are reduced by more than half. All values are well below international safety thresholds, making the recording room highly suitable for sensitive electrophysiological recordings. Gigahertz Solutions reports measurement uncertainties of ± 5% and ± 5 digital units for low-frequency fields (NFA1000) and ± 3 dB for high-frequency measurements (HF59B).

Although the laboratory is not subject to IEC 60601–1–2 medical device standards, limiting EMI is essential for high-precision biosignal recordings. Even though pre-installation EMI levels were already within safe limits and unlikely to cause major interference, additional shielding and grounding were implemented to further improve signal integrity. The recording room walls, ceiling, and door were coated with two layers of conductive paint (CEM YSHIELD HSF54), connected to a dedicated ground. Ventilation ducts were equipped with metallic grilles to prevent EMI leakage. To avoid generating additional EMI, data transmission inside the recording room uses grounded shielded cables. Cables between control and recording rooms are routed individually behind walls, supporting both electromagnetic isolation and orderly cable management (Fig. S2 in Supplementary Materials). Light transformers were placed as far as possible from the participant's position and enclosed in a metallic box above the ceiling to minimize EMI from the lighting system.

#### Environmental controls

To ensure appropriate experimental conditions and participant comfort, the laboratory uses a centralized HVAC system that maintains temperature between 20 and 22 °C and relative humidity between 40 and 60%, in line with recommendations for experimental psychology and cognitive neuroscience research. A thermometer/hygrometer (NOKLEAD) was installed in both the recording and control rooms to continuously monitor local conditions (see Table 7 for technical specifications). Continuous monitoring helps prevent environmental fluctuations that could compromise data quality or participant comfort during experiments.

To quantify real operational conditions, multiple instruments were used to monitor air quality, thermal parameters, and atmospheric pressure over a 19-min interval with 30-s sampling. A hot-wire anemometer measured airflow, temperature, and pressure, while a CO_2_ sensor recorded temperature, humidity, pressure, and CO_2_ concentration. A pressure monitor (Testo 400) provided absolute and differential pressure measurements. VOCs were measured using an Aeroqual Series 500, detecting compounds at 0.007 ppm, and formaldehyde reached 0.09 ppm. Surface temperatures of the floor, walls, and ceiling were uniform at 23 °C. These results confirm environmental stability and appropriate air quality, supporting consistent experimental conditions and participant comfort.

#### Lighting

The laboratory lighting system was designed to support participant comfort and cognitive performance while remaining compatible with neurophysiological recordings. In the recording room, neutral color temperatures around 4000 K were selected to provide good visual clarity and reduce visual fatigue. A modular DALI-based system was installed, allowing precise control of illuminance and distribution.

Lighting configuration was first simulated in DIALux and then validated with in situ measurements using a lux meter (ATP, ST-3809 LED). Measurements were collected at three positions: above the two participant tables and at an equivalent height in the center of the room. These data informed the final calibration of the lighting system (Table 6). Three experimental lighting scenarios were defined at 100%, 70%, and 50% intensity. The 70% setting, providing approximately 400 lx at the participant position, was selected as the default condition for experiments, balancing comfort and task visibility. The 100% setting is used for setup tasks and general room preparation, while the 50% setting is reserved for tasks requiring reduced illumination, such as meditation or mindfulness-based interventions.

**Table 6:** Summary of light intensity measurements (in lux) at three positions in the recording room (left participant station, right participant station, and middle of the room) under three lighting conditions (50%, 70%, and 100%)

	Position	Light intensity at 100% (lux)	Light intensity at 70% (lux)	Light intensity at 50% (lux)
Position 1	Participant station (left)	2,162	393	144
Position 2	Participant station (right)	2,273	413	150
Position 3	Middle of the room	780	396	135

All measurements were performed at a height of 75 cm.

In addition to direct illumination, reflectance was assessed to ensure uniform distribution and limit glare. Under the 70% condition, reflected light was measured as 71 lx on the table, 35 lx on the wall opposite the participant, 12 lx on the floor, and 29 lx on the ceiling. These values indicate that material reflectance supports adequate light diffusion without excessive glare, providing a visually comfortable environment while maintaining sufficient optimal visibility for experimental tasks.

### Acquiring and integrating research equipment

#### General lab equipment

The selection of general laboratory equipment was guided by the need for precise stimulus presentation, accurate response recording, robust participant monitoring, and secure data handling while maintaining stable environmental conditions. The resulting setup is versatile and can be adapted to a wide range of experimental paradigms. The main devices are summarized in Table 7 and provide a comprehensive infrastructure for controlled experimentation and high-quality data acquisition.

**Table 7:** Selected general laboratory equipment for the multimodal cognitive neuroscience laboratory

Item	Company & model	Key features & specifications	Purpose in the lab
Monitor	Tobii Pro Spectrum Display	- 24.5-inch FHD display (1920 × 1080) - 300 Hz refresh rate - Low-latency response for precise stimulus presentation	Visual stimulus presentation with high refresh rate for cognitive experiments with eye-tracking
Microphone (participant)	Logitech Blue Yeti	- USB condenser microphone - Multiple polar patterns - Plug-and-play design	High-quality voice recordings for behavioral tasks, voice analyses, and voice cloning
Over-ear headphones (participant)	AKG K-182	- Over-ear design - Frequency response: 10 Hz–28 kHz - Closed-back for noise isolation	Auditory stimulus presentation in tasks not involving EEG/fNIRS, providing minimal external interference
In-ear headphones (participant)	Shure SE215	- In-ear design - Frequency response: 22 Hz–17.5 kHz - Passive noise isolation up to ~ 37 dB	Auditory stimulus presentation during EEG/fNIRS recordings, avoiding mechanical interference with electrodes and minimizing artifact risk
Response pad	Black Box Toolkit USB Response Pad	- Custom 8-button layout - 24 mm tactile buttons - Ultra-low latency USB connection - Laser-cut custom button configuration	Precise reaction time measurement and participant response recording
Surveillance camera	Synology BC500	- 5 MP resolution (2880 × 1620) - Night vision - AI-powered motion detection	Participant monitoring during experiments
Webcam	OSBOT Tiny 2 Lite	- 4 K UHD resolution - AI auto-tracking - HDR support	Facial expression analysis and researcher–participant communication
Ceiling speaker (recording room)	Audibax KA06 White	- 6.5-inch speaker - 100 W power output - Wide frequency response	Researcher–participant communication
Microphone (researchers)	Proel BMG2	- Dynamic microphone - Cardioid polar pattern - Balanced XLR output	Researcher–participant communication for instructions and participant guidance
Audio mixer amplifier	Adastra DM25	- Digital mixer amplifier - Bluetooth-enabled	Microphone amplification for researcher instructions and participant guidance
Network storage (NAS)	Synology DiskStation DS224 +	- Two-bay NAS system with RAID support - Intel® Celeron® J4125 processor - AES-NI hardware encryption - Automatic backup and secure data management	Secure storage for experimental data, ensuring compliance with GDPR & LOPDGDD
External audio interface	SSL 2 +	- 24-bit/192 kHz resolution - 2 XLR/line inputs - Low-latency monitoring	High-quality audio recording and processing
Wall-mounted speakers (researchers)	Presonus Eris 3.5 (2nd Gen)	- 3.5-inch woofer - Frequency range: 80 Hz – 20 kHz - Active nearfield monitors	High-fidelity sound output for auditory experiments
Thermometer/hygrometer	NOKLEAD White	- Measures temperature (−9.9ºC ~ 60ºC)—Measures humidity (10% ~ 95% RH) - Large LCD - Battery-powered	Environmental monitoring for optimal lab conditions

Summary of general laboratory equipment used for stimulus presentation, response collection, participant monitoring, and environmental control. The table details the company, product/model, key technical specifications, and purpose in the lab, emphasizing how each device contributes to experimental workflows and data acquisition.

A surveillance camera (Synology BC500) was installed in the recording room to enable real-time monitoring of participant behavior, particularly during hyperscanning experiments. High-resolution webcams (OSBOT Tiny 2 Lite) were added to capture facial expressions, supporting analyses of emotion and social interaction. These systems improve experimental control and behavioral annotation while complying with ethical requirements regarding privacy and data security.

Communication between researchers and participants is supported by ceiling-mounted speakers (Audibax KA06) in the recording room, driven by a Proel BMG2 microphone and an Adastra DM25 digital mixer amplifier. Wall-mounted speakers (Presonus Eris 3.5 2nd Gen) in the control room allow researchers to monitor participant responses clearly.

For speech-based tasks, verbal responses are recorded with a Logitech Blue Yeti microphone, selected for its flat response spectrum. Because microphones introduce frequency response variability, devices with relatively flat responses were prioritized to preserve the spectral characteristics of recorded speech. For paradigms requiring higher precision in voice quality, a dedicated cardioid condenser microphone is recommended.

For tasks that do not involve EEG or fNIRS, over-ear closed-back headphones (AKG K-182) are used to provide acoustic isolation and minimize sound leakage. During EEG/fNIRS recordings, low-profile in-ear monitors (e.g., Shure SE215) are used instead to avoid mechanical interference with the cap and reduce motion-related artifacts. All headphone models were chosen for their relatively flat frequency responses, minimizing spectral distortion and ensuring accurate auditory stimulus delivery. Given that room acoustics shape perceived sound, headphone-based delivery is preferred whenever stringent control of auditory stimulation is required. If further noise reduction becomes necessary, active noise-cancelling (ANC) headphones may be considered to mitigate external sounds.

Audio routing is managed through a USB audio interface (SSL 2 +), which supports high-fidelity sound processing and real-time communication between control and recording rooms. Loudspeaker output is calibrated to minimize room-induced coloration in spoken instructions and auditory stimuli.

Visual stimuli are presented on a Tobii Pro Spectrum monitor, providing a 24.5-inch full high-definition (FHD) display with a 300-Hz refresh rate. This configuration ensures low-latency, high-refresh visual presentation suitable for cognitive, perceptual, and reaction time experiments.

Behavioral responses are collected with a customizable eight-button USB response pad (The Black Box Toolkit). The laser-cut layout was tailored to the laboratory’s experiments, with seven buttons arranged in an arc for Likert-scale responses and an additional button in the lower center position to mimic a keyboard space bar. This configuration provides intuitive input and high-precision reaction time measurement. Wired keyboards and mice are also available, eliminating Bluetooth interference and reducing timing variability in high-precision tasks.

#### Research equipment

The core recording systems used in the laboratory are listed in Table 8 and provide a flexible platform for multimodal cognitive neuroscience research. These devices were selected for their configurability, integration capabilities, and precise synchronization, supporting both single-participant and hyperscanning designs with consistent temporal and spatial resolution across modalities.

**Table 8:** Summary of the selected multimodal cognitive neuroscience laboratory recording equipment

Equipment type	Company & model	Key features & specifications	Multimodal integration
EEG amplifier	BrainProducts actiChamp Plus	- Sampling rate: up to 100 kHz - Channel configuration: up to 160 channels - Auxiliary inputs: supports up to eight peripheral biosensors - Low-noise amplifier for high signal fidelity	- Wired TTL synchronization for multimodal and hyperscanning experiments via Trigger Box
EEG cap	BrainProducts actiCap	- Electrode type: compatible with wet and active electrodes - Configurable layouts for flexible electrode placement - Lightweight and comfortable design for long recordings	- Fully compatible with actiChamp Plus amplifier - Compatible with NIRx (NIRSport2) optodes for fNIRS-EEG co-recording
Trigger synchronization	BrainProducts Trigger Box Plus	- Precise event marking system for EEG, fNIRS, and external devices - Low-latency TTL signal triggering	- Enables synchronized multimodal recordings (EEG, fNIRS, eye-tracking, electrical stimulator, peripheral biosensors)
Electrode location scanner	BrainProducts CapTrak	- Camera-based EEG electrode localization - 3D head model reconstruction for improved source localization	Not applicable
Peripheral biosensors (GSR, ExG, PPG, accelerometer, respiration belt)	BrainProducts	- Seamless integration with actiChamp amplifier - Multimodal physiological signal acquisition	- Direct integration with EEG system (actiChamp Plus)
fNIRS	NIRx NIRSport2	- Sampling rate: up to 240 Hz - Channel configuration: up to 256 optodes - Adjustable source–detector distances for optimized signal quality	- Wired TTL synchronization for multimodal and hyperscanning experiments - Compatible with actiCap EEG cap for simultaneous fNIRS-EEG recordings
Eye tracker	Tobii Pro Spectrum	- Sampling rate: up to 1200 Hz - Accuracy: 0.17° - Resolution (RMS): 0.25° without head support - Latency: < 3 ms (end-to-end delay)	- TTL synchronization for real-time integration with other systems
Electrical stimulator	Digitimer DS7A	- Pulse duration: 50 µs to 2 ms - Current output: 100 mA (constant) from 400 V - Designed for controlled neuromodulation studies	- TTL-compatible trigger options for precise timing in multimodal setups

Overview of the recording equipment chosen for advanced cognitive neuroscience research, including EEG, fNIRS, eye-tracking, peripheral biosensors, and electrical stimulation systems. The table details the company, product/model, key features, and multimodal capabilities, highlighting integration, synchronization, and high-performance specifications to support multimodal and hyperscanning experiments.

For EEG, the laboratory uses two actiChamp Plus amplifiers (BrainProducts) and a set of actiCap EEG caps in multiple sizes. The flexible channel configuration allows customized montages adapted to specific experimental protocols. An EEG electrode localization scanner (BrainProducts CapTrak) is used to capture three-dimensional electrode positions, improving spatial accuracy and source reconstruction (Eom, 2022).

A comprehensive set of peripheral biosensors (BrainProducts) supports multimodal physiological monitoring, including GSR, ECG, photoplethysmography (PPG), respiration belts, and accelerometers. These sensors integrate directly with the EEG amplifiers, ensuring synchronous acquisition of central and peripheral physiological signals. The configuration allows simultaneous recordings from multiple participants, supporting interactive paradigms and hyperscanning studies.

The selected fNIRS system (NIRSport2, NIRx) complements the EEG setup by providing hemodynamic measurements that can be co-registered with electrophysiological data. The system is designed for scalability and can be expanded with additional modules as future research demands increase.

An electrical stimulator (Digitimer DS7A) is included for studies of somatosensation, sensory integration, and fear conditioning. It delivers controlled electrical pulses to probe somatosensory and neuromodulatory mechanisms.

Eye movements are recorded with an eye-tracking system (Tobii Pro Spectrum), enabling high-speed sampling and low-latency gaze-tracking. The system is suited to language, reading, and cognitive tasks and can be synchronized with EEG and fNIRS for multimodal analyses.

Temporal alignment across devices is managed with a trigger synchronization box (BrainProducts Trigger Box Plus), which distributes event markers across acquisition systems and supports precise multimodal synchronization. In hyperscanning configurations, EEG, fNIRs, and peripheral biosensor amplifiers are synchronized via TTL connections to ensure consistent timing across participants and modalities.

#### Data management and compliance

The laboratory operates under European and Spanish ethical and legal frameworks, including Regulation (EU) 2016/679 (GDPR, 2016) and the Spanish Organic Law 3/2018 on Data Protection and Digital Rights (LOPDGDD, 2018). Data collection involves multiple streams, including biosignals (EEG, fNIRS, peripheral biosensors), behavioral measures (reaction times, motor responses), questionnaire data, and audiovisual recordings (surveillance cameras, webcam footage). All research data is stored on a centralized NAS (Synology DiskStation DS224 +), which provides secure multi-user access, automated backups, and encrypted storage (see Table 7 for technical specifications). This infrastructure ensures that data remains safe, compliant, and accessible while protecting participant confidentiality.

Consent documentation is stored securely to demonstrate informed consent for data collection, processing, and potential publication. Physical forms are kept in locked storage with restricted access, and scanned copies are digitally archived on the NAS with encryption and role-based access controls. Because the laboratory works with both adults and children, parental or legal guardian consent is obtained for minors in line with GDPR requirements for child data protection (GDPR, 2016; Article 8).

Given the sensitivity of human research data, additional security measures are in place. All digital records—including biosignals, questionnaires, and audiovisual files—are encrypted using AES-256. Remote access to the NAS requires multi-factor authentication (MFA), reducing the risk of unauthorized access. In accordance with GDPR guidelines, participants' data is stored for at least 5 years after publication. After this period, data are either anonymized for potential use or securely deleted.

Data loss prevention relies on a structured backup strategy. Automated weekly backups provide redundancy and enable rapid recovery in the event of accidental deletion, hardware failure, or cyber incidents. A secure off-site backup is maintained in a separate, access-controlled location to further protect long-term data integrity.

## General discussion

Developing a multimodal experimental psychology and cognitive neuroscience laboratory requires the coordinated integration of technological, environmental, and methodological elements to ensure high-quality data acquisition and experimental reproducibility. Different research aims place different demands on the environment—for example, tight lighting control is essential in visual perception studies, while VOC management is mandatory in olfactory experiments. A well-designed laboratory must therefore remain flexible, supporting diverse paradigms while maintaining rigorous methodological control to ensure reliable outcomes.

Ensuring multimodal research hinges on the strategic selection and integration of recording technologies. Employing hardware designed for multimodal compatibility—such as EEG-fNIRS hybrid caps or amplifiers with dedicated synchronization channels—reduces the need for external timing systems and improves temporal alignment across physiological signals. Electromagnetic shielding and noise-reduction measures are equally essential for preserving data quality. Implementing Faraday cages, proper grounding, and shielded cabling substantially reduces EMI and improves signal fidelity for EEG and fNIRS. In parallel, robust data security measures—including NAS-based storage, AES-256 encryption, and multi-factor authentication—safeguard long-term data integrity and ensure compliance with GDPR (2016) and LOPDGDD (2018) regulations.

Environmental control plays a similarly critical role. The acoustic treatments implemented in the laboratory effectively mitigated room eigenvalues, improving speech quality and reducing ambient noise to below 37 dBA—an important threshold for minimizing interference in biosignal recordings. Stable air quality, characterized by low VOC and formaldehyde emissions and controlled CO_2_ levels, supports participant comfort and prevents sensor contamination. Lighting assessments confirmed uniform, diffuse illumination with minimal glare, providing a controlled visual environment for experiments requiring precise luminance regulation. Together with high-precision stimulus delivery systems, these environmental controls establish a research setting optimized for reproducible, high-quality data collection.

Synchronization remains one of the central challenges in multimodal neuroscience. Hardware-based solutions—TTL triggers, photodiodes, or dedicated event-marking devices—offer the most reliable temporal alignment across EEG, fNIRS, eye-tracking, and peripheral biosensors. When hardware solutions are not feasible, software frameworks such as LSL provide effective alternatives, enabling timestamp-based synchronization and post hoc alignment. Proper timing control is essential for preventing misalignment between modalities, which can lead to interpretational errors or systematic biases in multimodal datasets.

Standardization and transparent reporting are fundamental for reproducibility. Documenting laboratory settings, equipment specifications, synchronization strategies, and environmental conditions provides essential metadata for replication across studies and research teams. As multimodal research becomes more prevalent, widespread adoption of best practices in integration, data management, and methodological transparency will be key to producing findings that are robust, generalizable, and comparable across laboratories.

Ultimately, establishing standardized multimodal laboratory environments is about optimizing the SNR of biosignals to capture brain–behavior relationships with maximal fidelity. Variability in lighting, acoustic properties, electromagnetic shielding, or synchronization protocols introduces noise and reduces sensitivity to genuine neural effects. Equally important is the transparent documentation of these environmental and methodological conditions, as even subtle variations—such as monitor refresh rates, ambient noise levels, or lighting spectra—can influence cognitive and neural responses. Systematic reporting of equipment parameters, environmental measurements, and experimental protocols provides critical metadata for replication and enhances comparability across studies, enabling higher-fidelity reproduction and cumulative scientific progress.

To support this goal, we propose the development of an index that quantitatively characterizes laboratory environments using measurable parameters. Such an index could condense key variables—lighting, EMI shielding, acoustic treatment, air quality, and synchronization precision—into a standardized metric to report laboratory conditions systematically. Incorporating this index into publications would improve methodological transparency and facilitate cross-study comparisons.

Standardized environments also make it possible to tailor laboratories to specific research domains—such as visual processing, language, or social cognition—while preserving consistency in stimulus delivery, response recording, and environmental control. This supports replication, meta-analyses, and large-scale data sharing initiatives, ultimately advancing the field toward more generalizable models of brain function.

In sum, establishing a multimodal cognitive neuroscience laboratory bridges the gap between theoretical aims and empirical implementation, providing controlled, reproducible, and high-fidelity environments essential for experimental psychology and cognitive neuroscience.

## Conclusions

This work demonstrates the feasibility of establishing a multimodal experimental psychology and cognitive neuroscience laboratory that integrates advanced neuroimaging and biosignal recording technologies under rigorous environmental control. By implementing targeted acoustic treatment, electromagnetic shielding, precise synchronization strategies, and optimized air quality and lighting conditions, the laboratory provides a standardized infrastructure capable of supporting high-fidelity EEG, fNIRS, eye-tracking, and peripheral biosensor recordings. These design decisions markedly enhance data quality, reduce external interference, and improve SNR—critical factors for accurately capturing brain–behavior relationships.

Beyond the technological aspects, this work highlights the importance of methodological transparency and standardization. Detailed documentation of experimental protocols, environmental conditions, and recording procedures facilitates replication across studies and supports the development of benchmark laboratory standards for diverse applications in experimental psychology and cognitive neuroscience. Standardized control over ambient noise, lighting, and air quality contributes to more reliable and comparable findings across laboratories.

The framework presented here serves as a model for future neuroscience laboratories, demonstrating how thoughtful design, equipment selection, and environmental optimization can support scalable and reproducible research. As multimodal cognitive neuroscience continues to evolve, future efforts will likely focus on integrating emerging technologies, refining synchronization methods, and expanding experimental paradigms. Establishing shared best practices for multimodal integration, environmental control, and data management will be essential for advancing rigorous, reproducible experimental psychology and cognitive neuroscience research.

## Supplementary Information

Below is the link to the electronic supplementary material.

Supplementary file1 (1.41 MB)

## Data Availability

All data and materials supporting this study are available on the Open Science Framework (OSF) at https://osf.io/vcs8u/. The repository includes datasets and scripts related to the laboratory’s acoustic treatment measurements, electromagnetic interference (EMI) analyses, and lighting system simulations and measurements, as well as documentation describing the procedures and results.

## Footnotes

1 In some cases, multiple recording rooms may be considered, depending on factors such as available space, budget, and the number of concurrent experiments. However, additional recording rooms substantially increase costs associated with soundproofing, EMI shielding, licensing, and equipment maintenance. A more cost-effective approach is to begin with one recording room and one control room, ensuring efficient use of resources while maintaining high experimental standards.

2 Setup time varies considerably, depending on the number of electrodes, the conductive medium used, impedance-checking procedures, and researcher’s training expertise.

3 According to the manufacturer NIRx, recent technical advances in high-density fNIRS allow spatial resolution comparable to fMRI, along with higher sampling rates. Retrieved March 28, 2025, from https://nirx.net.

4 Approximate costs vary depending on laboratory dimensions, material quality, and desired performance. As a reference, acoustic treatment ranges from €5,800 (basic absorption without isolation) to €26,000 (with full isolation), while electromagnetic shielding ranges from €6,400 (≈40 dB attenuation) to €180,000 (80–100 dB attenuation with low-frequency reinforcement). These values are indicative and may vary by supplier and regional labor costs.

## Code availability

The Python scripts used in this study for data processing and figure generation in the acoustic treatment and electromagnetic interference (EMI) analyses are available via the same OSF repository (https://osf.io/vcs8u/).
