# Supplementary material for: From zero to lab: Guidelines and practical implementation for building a multimodal experimental psychology and cognitive neuroscience laboratory
Source: Behav Res Methods. 2026 Mar 30;58(4):98. doi: 10.3758/s13428-026-02988-0 (PMC13035650; doi:10.3758/s13428-026-02988-0)
Supplement: Supplementary file 1 — Supplementary file1 (1.41 MB) [file 13428_2026_2988_MOESM1_ESM.docx]

# Supplementary materials

## Recording equipment

**Tables S1-8** summarize commercially available EEG, fNIRS, eye-tracking, and peripheral biosensor systems. Beyond the modality-specific characteristics of each device, several cross-cutting features strongly influence system suitability for multimodal research. These include multimodal integration capabilities, synchronization options, hyperscanning support, portability, customization, and compatibility with common operating systems and analysis platforms. All specifications were compiled from manufacturers’ official documentation and product websites.

###### TABLE S1 | Comparison of commercial EEG systems, including specifications such as number of channels, sensor types, resolution, sampling rate, bandwidth, common-mode rejection ratio (CMRR), and input impedance. Data sourced from respective manufacturers' websites.

| **Device** | **Company** | **Channels** | **Sensors type** | **Resolution** | **Sampling rate** | **Bandwidth** | **CMRR** | **Input Impedance** |
| --- | --- | --- | --- | --- | --- | --- | --- | --- |
| Versatile EEG | BitBrain | 8 - 64 | Semi-dry, active shielding | 24 bits | 256 Hz | DC to 70 Hz | > 100 dB @ 50Hz | >50 GΩ |
| Enobio | Neuroelectrics | 8 - 32 | dry & wet | 24 bits | 500 Hz | DC to 125 Hz | 115 dB | ≥ 1 GΩ |
| GES 400 | Geodesic | 32 - 256 | Wet, active | 24 bits | up to 8 kHz | DC to 2 kHz | ≥ 90 dB | ≥ 1 GΩ |
| Active three | Biosemi | 16-136 | Wet, active | 24 bits | up to 16 kHz | DC to 5.4 kHz | > 100 dB @ 50 Hz | 600 MOhm @ 50 Hz |
| ActiCHamp plus | BrainProducts | 32 - 160 | Wet, active | 24 bits | 25 to 100 kHz | DC to 7,5 kHz | 100 dB | ≥ 1 GΩ |
| eego™ mylab | ANTneuro | 32 - 256 | Dry, semi-dry & wet, active shielding | 24 bits | up to 16 kHz | DC to 4.2 kHz | > 100 dB | ≥ 1 GΩ |
| g.HIAMP | g.tec | 16 - 256 | Wet, active/passive | 24 bits | 38.4 kHz | DC to 1 kHz | > 100 dB | ≥ 1000 GΩ |
| SAGA | TMSI | 32 - 128 | Dry, semi-dry & wet | 24 bits | up to 4 kHz | DC to 800 Hz | 100 dB @ 50Hz | > 1 GΩ |

######

###### TABLE S2 | Overview of commercial EEG systems with multimodal compatibility, synchronization capabilities, and communication methods. The table details auxiliary channels, platform compatibility, and whether systems are wired or wireless. Data is sourced from respective manufacturers' websites.

| **Device** | **Multimodal (brain activity)** | **Hyperscanning or synchronization capabilities** | **Auxiliary channels** | **Other integrated sensors** | **Platform & Device Compatibility** | **Wireless vs. Wired Communications** |
| --- | --- | --- | --- | --- | --- | --- |
| Versatile EEG | fNIRS | - LSL compatibility  - 1 digital input (1bit)  - 1 optical trigger | 6 bipolar auxiliary channels | Accelerometer, gyroscope, and magnetometer | - SDK for Windows and Linux (C/C++)  - Matlab and Python | Bluetooth (up to 10 meters) |
| Enobio | fNIRS, TMS, tES | - TTL triggers  - LSL and TCP/IP compatibility | No | No | - Windows and macOS  - SDK for Windows and Linux  - Matlab and Python | Wi-Fi IEEE 802.11g or USB |
| GES 400 | fMRI, MEG, TMS | - Built-in clock sync  - TTL triggers | Support for 32 extra channels | No | - macOS  - SDK for developers  - Matlab and Python | Fiber optics / USB |
| Active three | Info not provided | TTL triggers | 6 bipolar auxiliary channels | No | - Windows, Linux and macOS  - SDK for Windows, Linux and macOS  - Matlab and Python | Fiber optics / USB |
| ActiCHamp plus | fNIRS, fMRI, MEG, TMS, tES | Additional module for TTL triggers | 8 auxiliary channels | No | - Windows  - Matlab and Python | USB |
| eego™ mylab | TMS, MEG, tES | - TTL triggers  - LSL compatibility | Up to 96 bipolar channels | No | - Windows  - SDK for Windows and Linux  - Matlab | USB |
| g.HIAMP | fNIRS, TMS, tES | - TTL triggers  - LSL compatibility | 16 auxiliary channels | No | - Windows  - SDK for Windows  - Matlab and Python | USB |
| SAGA | fNIRS, TMS | - Sync module  - LSL compatibility | 9 auxiliary channels | No | - Windows and Linux  - Matlab and Python | Fiber optics / USB |

######

###### TABLE S3 | Comparison of commercial fNIRS systems, highlighting key features such as the number and type of light sources, wavelengths, number of detectors, source-detector distance, optode configuration, and sampling rate. Data sourced from respective manufacturers' websites.

| **Device** | **Company** | **Num of sources** | **Source light type** | **Source wavelengths** | **Num of detectors** | **Source-detector distance** | **Optode type** | **Sampling rate** |
| --- | --- | --- | --- | --- | --- | --- | --- | --- |
| Brite Family | Artinis | up to 54 channels | LED | 760 and 850 nm | up to 54 channels | 10 to 55 mm | Modular | up to 75 Hz |
| Photon Cap C20 | Cortivision | 16 | LED | 760 and 850 nm | 10 | around 30 mm | Modular | up to 86 Hz |
| g.SENSOR fNIRS | g.tec | 8 | LED (high/low power options) | 760 and 850 nm | 2 | around 30 mm | Modular | 10 Hz |
| NIRScout | NIRx | 8 to 64 | LED, Laser | LED: 760 & 850nm Laser: 785, 808, 830, & 850nm | 4 to 32 | Adjustable (up to 7.5 cm) | Modular | 100 Hz |
| NIRSport2 | NIRx | 8 to 80 | LED | LED: 760 & 850 nm | 8 to 80 | Adjustable (by default is 8 mm) | Modular | up to 240 Hz |
| MedelOpt | BIOPAC Systems | 16 | LED | 730 and 850 nm | 32 | 20 to 55 mm | Modular | up to 64 Hz |
| Nirsit | OBELAB | 24 | LED | 780 and 850 nm | 32 | 1.5 to 3.35 cm | Embedded | 8.138 Hz |
| Flow2 | Kernel | 3 | LED | 690 and 90 5nm | 6 | 8.5 to 60 mm | Modular | 3.76Hz |

######

###### TABLE S4 | Comparison of commercial fNIRS systems, highlighting multimodal compatibility, wireless and wired configurations, hyperscanning capabilities, and synchronization options. Data sourced from respective manufacturers' websites.

| **Device** | **Multimodal (brain activity)** | **Other integrated sensors** | **Wireless vs. Wired Systems** | **Hyperscanning or synchronization capabilities** | **Platform & Device Compatibility** |
| --- | --- | --- | --- | --- | --- |
| Brite Family | EEG, tES | Accelerometer, gyroscope | Bluetooth | Supports hyperscanning and synchronization | - Windows  - Matlab, Python |
| Photon Cap C20 | EEG | Accelerometer, gyroscope | Wireless | LSL protocol | - Windows, Linux, macOS  - Matlab, Python |
| g.SENSOR fNIRS | EEG | No | Wireless (10 meters indoor) | - TTL triggers  - LSL compatibility | - Windows  - SDK for Windows (C/++)  - Matlab, Python |
| NIRScout | EEG, fMRI, TMS, tES | No | Wired | - Up to 4 separate bi-lateral 16-source/8-detector arrays for 4 subjects  - TTL triggers  - LSL compatibility | - Windows, macOS  - Matlab |
| NIRSport2 | EEG, tES, MRI, MEG, TMS | Accelerometer | Wired/WiFi | - Up to 10 or more subjects  - LSL compatibility  - TTL triggers | - Windows, macOS  - Matlab |
| MedelOpt | EEG | No | Wired/ WiFi /Bluetooth | MedelOpt tandem (2 devices) | Windows, macOS |
| Nirsit | Info not provided | Accelerometer, gyroscope | USB/ Wireless | Synchronization with event markers | Windows |
| Flow2 | EEG | No | USB | Info not provided | - Linux, macOS  - SDK for developers |

######

###### TABLE S5 | Comparison of commercial eye-tracking systems, including wearable, remote, and head-supported configurations. Key features, such as accuracy, resolution, sampling rate, latency, and platform compatibility, are highlighted. Data is sourced from respective manufacturers' websites.

| **Device** | **Company** | **Type** | **Accuracy** | **Resolution** | **Sampling rate** | **Latency in real-time processing** | **Hyperscanning or synchronization capabilities** | **Platform and Device Compatibility** |
| --- | --- | --- | --- | --- | --- | --- | --- | --- |
| Tobii Pro Glasses 3 | Tobii | Wearable (glasses) | 0.6º | Info not provided | 50 or 100 Hz | Low latency (<10 ms) | No | Windows, macOS, Android |
| Neon | Pupil Labs | Wearable (glasses) | up to 1.3º | Info not provided | up to 200 Hz | Low latency | No | VR platforms, Windows, and macOS |
| Tobii Pro Spectrum | Tobii | Head-supported or remote | Down to 0.15º (Head supported)  Down to 0.17º (Head free-to-move) | Down to 0.01º RMS (Head supported)  Down to 0.25º RMS (Head free-to-move) | up to 1200 Hz | Extremely low latency (< 3 ms end-to-end delay) | TTL triggers | Windows |
| Eyelink 1000 plus | Eyelink | Head-supported or remote | Down to 0.15º (Head supported)  0.25º - 0.5º (Head free-to-move) | 0.01º RMS (Head supported)  0.05º RMS (Head free-to-move) | 2 kHz (Head supported)  1 kHz (Head free-to-move) | Extremely low latency (< 3 ms end-to-end delay) | Additional module for TTL triggers | Windows |
| Smart Eye Pro | Smart Eye | Remote | 0.5º | Info not provided | 60 Hz or 120 Hz | Low latency | Sync available | Windows |
| Smart Eye AI-X | Smart Eye | Remote | 0.5º | 0.1º | 60 Hz | Low latency | Info not provided | Windows |
| GP3 HD Eye Tracker | Gazepoint | Remote | 0.5º to 1º | Info not provided | 60 or 150 Hz | Moderate latency | Info not provided | Windows |

######

###### TABLE S6 | Overview of commercial peripheral biosensor systems, detailing channel configurations, signal quality, synchronization capabilities, communication methods (wired/wireless), and platform compatibility. Data is sourced from respective manufacturers' websites.

| **Device** | **Company** | **Channels** | **Signal quality and accuracy** | **Hyperscanning or synchronization capabilities** | **Wireless vs. Wired Systems** | **Platform and Device Compatibility** |
| --- | --- | --- | --- | --- | --- | --- |
| Versatile BIO | BitBrain | 21 channels (extensible to 35) | - Active shielding to improve SNR  - Sampling rate at 256 Hz | LSL protocol | Wireless (Bluetooth) or SD card | - Windows  - Matlab and Python |
| MP200 | BioPac | 16 channels | Sampling rate up to 100 kHz | - TTL triggers  - Sync via software | Ethernet | Windows and macOS |
| Research Ring | BioPac | No channels (integrated sensors) | Sampling rate up to 400 kHz | Sync via software | Wireless | Windows, iOS |
| BrainProducts Sensors | BrainProducts | 8 auxiliary channels in actiCHamp | Sampling rate up to 100 kHz | TTL triggers | USB | - Windows  - Matlab and Python |
| g.tec Sensors | g.tec | 16 channels (extensible module for g.HIAMP) | Sampling rate up to 38.4 kHz | - TTL triggers  - LSL compatible | Wireless or USB | - Windows ??  - SDK for Windows (C/++) for real-time  - Matlab and Python for post-processing |
| UHK-TA | iworx | 10 channels | Sampling rate up to 200 kHz | TTL triggers | Wired | Windows and macOS |
| Shimmer3/ Consensys | Shimmer | 4 channels | Sampling rate up to 8 kHz | Consensys platform | Wireless (Bluetooth) or SD card | Windows |
| TMSI Sensors | TMSI | 9 auxiliary channels in SAGA | Sampling rate up to 4 kHz | - Sync module  - LSL compatible | Wireless or USB/Ethernet | - Windows  - Matlab, Python, etc. for post-processing |

######

###### TABLE S7 | Biometrics measured in commercial peripheral biosensors systems (Part I). A dot (•) indicates that the corresponding biometric parameter is supported or measurable by the device listed in each row. Data sourced from respective manufacturers' websites.

| **Device** | **ECG** | **EMG** | **EOG** | **EGG** | **GSR/**  **EDA** | **AFS** | **Resp** | **Snore** | **Temp** | **PO** | **PPG/PG** | **BP** |
| --- | --- | --- | --- | --- | --- | --- | --- | --- | --- | --- | --- | --- |
| Versatile BIO | ᛫ | ᛫ | ᛫ |  | ᛫ | ᛫ | ᛫ | ᛫ | ᛫ |  | ᛫ |  |
| MP200 | ᛫ | ᛫ | ᛫ | ᛫ | ᛫ | ᛫ | ᛫ |  | ᛫ | ᛫ | ᛫ | ᛫ |
| Research Ring | ᛫ |  |  |  | ᛫ |  |  |  | ᛫ |  | ᛫ |  |
| BrainProducts Sensors | ᛫ | ᛫ | ᛫ |  | ᛫ |  | ᛫ |  |  |  | ᛫ |  |
| g.tec Sensors | ᛫ | ᛫ | ᛫ |  | ᛫ | ᛫ | ᛫ | ᛫ | ᛫ | ᛫ |  | ᛫ |
| UHK-TA | ᛫ | ᛫ |  |  | ᛫ | ᛫ |  |  | ᛫ | ᛫ | ᛫ | ᛫ |
| Shimmer3/ Consensys | ᛫ | ᛫ |  |  | ᛫ |  |  |  |  |  | ᛫ |  |
| TMSI Sensors | ᛫ | ᛫ | ᛫ |  | ᛫ |  | ᛫ |  | ᛫ | ᛫ | ᛫ |  |

###### TABLE S8 | Biometrics measured in commercial peripheral biosensors systems (Part II). A dot (•) indicates that the corresponding biometric parameter is supported or measurable by the device listed in each row. Data sourced from respective manufacturers' websites.

| **Device** | **Taylor** | **Grip/Load** | **Limb** | **Gonio** | **Steth** | **Photo** | **Accel** | **Gyro** | **Magnet** | **GPS** | **Altim** |
| --- | --- | --- | --- | --- | --- | --- | --- | --- | --- | --- | --- |
| Versatile BIO |  |  |  |  |  |  | ᛫ | ᛫ | ᛫ | ᛫ |  |
| MP200 |  | ᛫ |  | ᛫ | ᛫ |  | ᛫ |  |  | ᛫ |  |
| Research Ring |  |  |  |  |  |  | ᛫ |  |  |  |  |
| BrainProducts Sensors |  |  |  |  |  | ᛫ | ᛫ |  |  |  |  |
| g.tec Sensors |  |  | ᛫ |  |  |  | ᛫ |  |  |  |  |
| UHK-TA | ᛫ | ᛫ |  | ᛫ | ᛫ |  |  |  |  |  |  |
| Shimmer3/ Consensys |  |  |  |  |  |  | ᛫ | ᛫ | ᛫ |  | ᛫ |
| TMSI Sensors |  | ᛫ |  |  |  |  | ᛫ |  |  |  |  |

##

## Software solutions

**Table S9** provides an overview ofwidely used software tools for multimodal neuroscience research, including their capabilities for multimodal integration, statistical analysis, real-time versus offline processing, and platform compatibility. To ensure broad applicability, proprietary software tied exclusively to specific hardware manufacturers was not included; the focus is instead on open-source or widely compatible solutions. Additional tools for experiment design, stimulus presentation, and participant management are also listed, as these form an essential part of the cognitive neuroscience research workflow..

###### **TABLE S9 | Comparison of commercial post-processing software tools for neuroscience research**, highlighting their capabilities in multimodal integration, statistical analysis, real-time vs. post-processing functionality, and platform/OS compatibility. Note that although some software packages are free or open-source, they may require licensed environments (e.g., MATLAB) to operate. Data is sourced from manufacturers' official documentation and websites.

| **Software** | **Company** | **Modality focus** | **Multimodal integration** | **Statistical Analysis** | **Real-time** | **Post-**  **processing** | **Requirements** | **OS Compatibility** |
| --- | --- | --- | --- | --- | --- | --- | --- | --- |
| **EEGLAB toolbox** | Open-source | EEG | Partially, with plugins | Advanced | Limited, with plugins | Mainly | Matlab toolbox | Windows, Linux, and macOS |
| **ERPLAB toolbox** | Open-source | EEG | Partially, it relies on EEGLAB | Advanced, it relies on EEGLAB | No | Yes | Matlab toolbox | Windows, Linux, and macOS |
| **FieldTrip toolbox** | Open-source | EEG | Advanced | Advanced | Possible, with advanced configuration | Mainly | Matlab toolbox | Windows, Linux, and macOS |
| **MNE-Python** | Open-source | EEG | Advanced | Advanced | Possible, with external integration | Mainly | Python | Windows, Linux, and macOS |
| **OpenViBE** | Open-source | EEG (BCI) | EMG, eye-tracking | Basic | Mainly | Limited | Standalone | Windows, Linux |
| **Neuroguide** | Applied Neuroscience | EEG | No | Clinical statistics | Limited | Mainly | Standalone | Windows |
| **BESA** | BESA GmbH | EEG | MEG, fMRI | Advanced | No | Yes | Standalone | Windows |
| **Brainstorm** | Open-source | EEG | MEG, fMRI | Advanced | Limited, with plugins | Mainly | Matlab toolbox and standalone | Windows, Linux, and macOS |
| **NIRS toolbox** | Open-source | fNIRS | Partially, with plugins | Advanced | Limited, with plugins | Mainly | Matlab toolbox | Windows, Linux, and macOS |
| **Homer3** | Open-source | fNIRS | Peripheral biosensors | Advanced | No | Yes | Matlab toolbox | Windows, Linux, and macOS |
| **Ogama** | Open-source | Stimuli and eye-tracking | No | Basic | No | Yes | Standalone | Windows |
| **PyGaze** | Open-source | Eye-tracking | EEG | Basic, it relies on Python | Mainly | Limited | Python | Windows, Linux, and macOS |
| **ECG-kit** | Open-source | ECG | EEG and peripheral biosensors | Basic, it relies on Matlab | Limited | Mainly | Matlab toolbox | Windows, Linux, and macOS |
| **NeuroPype** | Intheon | Multimodal | EEG, fNIRS, ExG, eye-tracking, biosensors | Advanced | Yes | Yes | Standalone | Windows, Linux |
| **iMotions** | iMotions | Multimodal | EEG, fNIRS, ExG, eye-tracking, biosensors, etc | Advanced | Yes | Yes | Standalone | Windows |
| **The Observer XT** | Noldus | Multimodal and behavioral | EEG, eye-tracking, GSR, facial expression, heart rate, etc | Advanced | Yes | Yes | Standalone | Windows |
| **E-Prime** | Psychology Software tools | Behavioral and stimuli presentation | EEG, fNIRS, ExG, eye-tracking, biosensors | Basic | Mainly | Limited | Standalone | Windows |
| **PsychoPy** | Open-source | Stimuli and experiment design | EEG, fNIRS, ExG, eye-tracking, biosensors | Basic, it relies on Python | Mainly | Limited | Python | Windows, Linux, and macOS |
| **HyPyP** | Open-source | Hyperscanning | EEG, MEG, fNIRS | Advanced | No | Yes | Python | Windows, Linux, and macOS |

##

## Practical application: INAB Laboratory

### Acoustic attenuation

To optimize the acoustic properties of the laboratory, fiberglass soundproofing material was installed within the walls. In addition, 9-mm-thick acoustic panels made from biocompatible and sustainable materials (EcoCero, V-cut) were mounted on the room surfaces to improve acoustic comfort and reduce external noise infiltration (del Rosario-Gilabert et al., 2024). The Noise Reduction Coefficient (NRC) values for these panels are reported in **Table S10**.

###### TABLE S10 | Noise Reduction Coefficient (NRC) values NRC of acoustic panels mounted on the walls (EcoCero).

| **Octave band center frequencies [in Hz]** | **NRC reduction value** |
| --- | --- |
| 125 | 0.66 |
| 250 | 0.76 |
| 500 | 0.60 |
| 1000 | 0.80 |
| 2000 | 0.89 |
| 4000 | 0.80 |
| **Mean** | **0.75** |

### Electromagnetic shielding

A comprehensive electromagnetic interference (EMI) assessment was conducted to characterize and minimize EMI within the laboratory. Measurements were taken at two locations –one on each side of the room– at a height of 1.1 meters, using a three-minute acquisition window to evaluate emissions from existing electrical installations. **Table 5** in the main manuscript summarizes the peak electric field strength, magnetic flux density, and power density recorded prior to the laboratory setup. A peak electric field of 15.3 V/m was observed at 132.6 kHz (**Figure S1**), well below the 87 V/m reference level established by ICNIRP guidelines [(ICNIRP, 1998)](https://www.zotero.org/google-docs/?6MjF6E) for this frequency range.


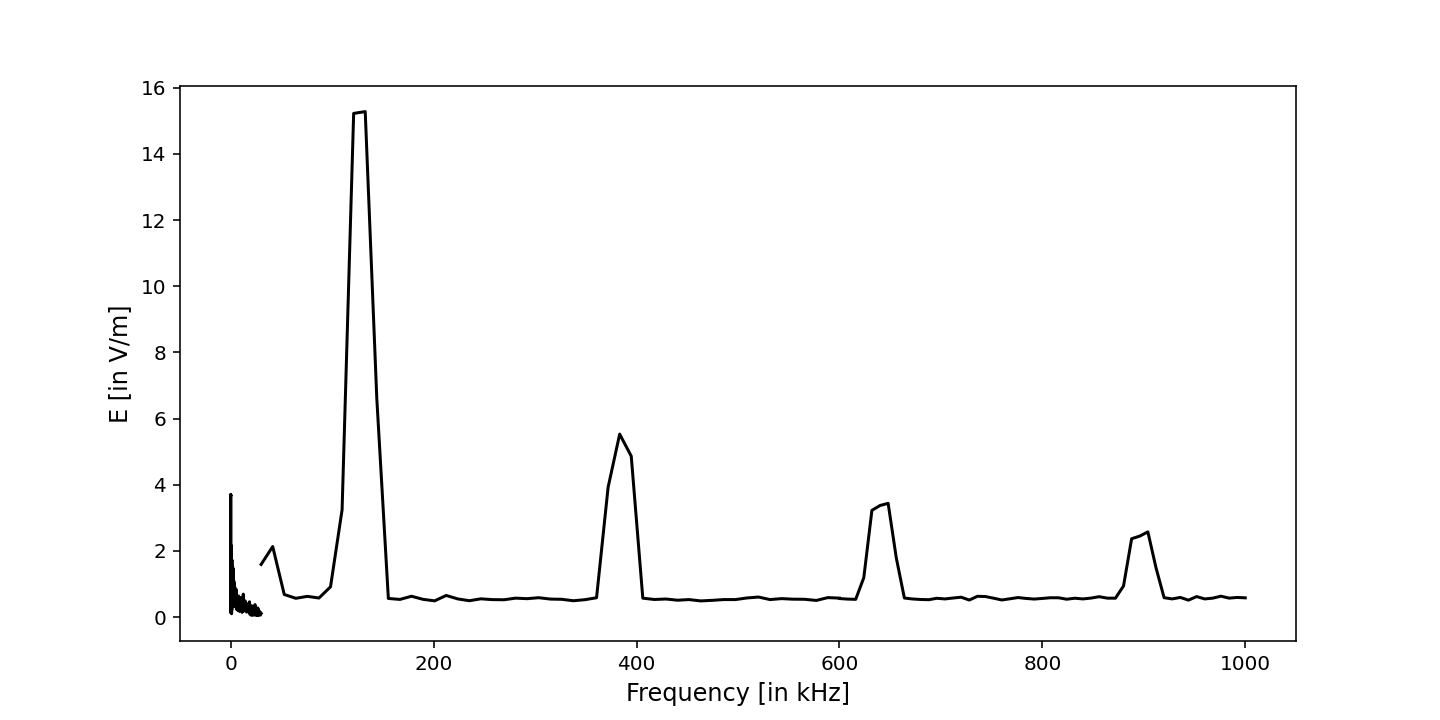


###### **FIGURE S1 | Electromagnetic spectrum measured in the laboratory before** the EMI shielding (recording room**).** The electric field (E) levels [in V/m] are plotted as a function of frequency [in kHz], with the highest peak recorded at 132.6 kHz at 15.3 V/m. These measurements provide a baseline for assessing potential EMI after installing the recording equipment.

To prevent the introduction of additional EMI during everyday operation, all data transfer within the recording room is performed through properly grounded, shielded wired connections. Cables were routed individually behind the walls to maintain an organized cabling system between the control and recording rooms and to further enhance electromagnetic isolation (**Figure S2**).


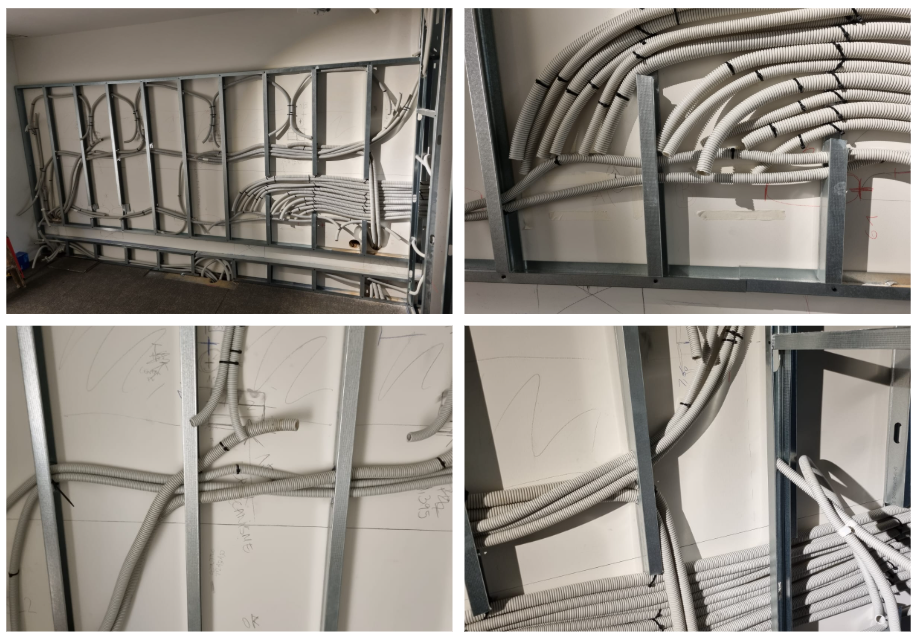


###### FIGURE S2 | Electromagnetic interference (EMI) reduction through shielded cabling. To minimize EMI, wired data transfer was implemented in the control and recording rooms using properly grounded, shielded cables, ensuring reliable and interference-free communication. Cables were routed individually behind the walls, maintaining an organized cabling system between the control and recording rooms while enhancing electromagnetic isolation.
